# Migraine Aura, Transient Ischemic Attacks, Stroke, and Dying of the Brain Share the Same Key Pathophysiological Process in Neurons Driven by Gibbs–Donnan Forces, Namely Spreading Depolarization

**DOI:** 10.3389/fncel.2022.837650

**Published:** 2022-02-10

**Authors:** Coline L. Lemale, Janos Lückl, Viktor Horst, Clemens Reiffurth, Sebastian Major, Nils Hecht, Johannes Woitzik, Jens P. Dreier

**Affiliations:** ^1^Center for Stroke Research Berlin, Berlin Institute of Health, Charité – Universitätsmedizin Berlin, Corporate Member of Freie Universität Berlin, Humboldt-Universität zu Berlin, Berlin, Germany; ^2^Department of Experimental Neurology, Berlin Institute of Health, Charité – Universitätsmedizin Berlin, Corporate Member of Freie Universität Berlin, Humboldt-Universität zu Berlin, Berlin, Germany; ^3^Department of Medical Physics and Informatics, University of Szeged, Szeged, Hungary; ^4^Department of Neurology, University of Szeged, Szeged, Hungary; ^5^Department of Neurology, Berlin Institute of Health, Charité – Universitätsmedizin Berlin, Corporate Member of Freie Universität Berlin, Humboldt-Universität zu Berlin, Berlin, Germany; ^6^Department of Neurosurgery, Berlin Institute of Health, Charité – Universitätsmedizin Berlin, Corporate Member of Freie Universität Berlin, Humboldt-Universität zu Berlin, Berlin, Germany; ^7^Department of Neurosurgery, Evangelisches Krankenhaus Oldenburg, University of Oldenburg, Oldenburg, Germany; ^8^Bernstein Center for Computational Neuroscience Berlin, Berlin, Germany; ^9^Einstein Center for Neurosciences Berlin, Berlin, Germany

**Keywords:** migraine aura, traumatic brain injury, circulatory arrest, subarachnoid hemorrhage, spreading depolarization, spreading depression, brain death, brain ischemia

## Abstract

Neuronal cytotoxic edema is the morphological correlate of the near-complete neuronal battery breakdown called spreading depolarization, or conversely, spreading depolarization is the electrophysiological correlate of the initial, still reversible phase of neuronal cytotoxic edema. Cytotoxic edema and spreading depolarization are thus different modalities of the same process, which represents a metastable universal reference state in the gray matter of the brain close to Gibbs–Donnan equilibrium. Different but merging sections of the spreading-depolarization continuum from short duration waves to intermediate duration waves to terminal waves occur in a plethora of clinical conditions, including migraine aura, ischemic stroke, traumatic brain injury, aneurysmal subarachnoid hemorrhage (aSAH) and delayed cerebral ischemia (DCI), spontaneous intracerebral hemorrhage, subdural hematoma, development of brain death, and the dying process during cardio circulatory arrest. Thus, spreading depolarization represents a prime and simultaneously the most neglected pathophysiological process in acute neurology. Aristides Leão postulated as early as the 1940s that the pathophysiological process in neurons underlying migraine aura is of the same nature as the pathophysiological process in neurons that occurs in response to cerebral circulatory arrest, because he assumed that spreading depolarization occurs in both conditions. With this in mind, it is not surprising that patients with migraine with aura have about a twofold increased risk of stroke, as some spreading depolarizations leading to the patient percept of migraine aura could be caused by cerebral ischemia. However, it is in the nature of spreading depolarization that it can have different etiologies and not all spreading depolarizations arise because of ischemia. Spreading depolarization is observed as a negative direct current (DC) shift and associated with different changes in spontaneous brain activity in the alternating current (AC) band of the electrocorticogram. These are non-spreading depression and spreading activity depression and epileptiform activity. The same spreading depolarization wave may be associated with different activity changes in adjacent brain regions. Here, we review the basal mechanism underlying spreading depolarization and the associated activity changes. Using original recordings in animals and patients, we illustrate that the associated changes in spontaneous activity are by no means trivial, but pose unsolved mechanistic puzzles and require proper scientific analysis.

## Introduction

The brain is the most complex structure known in the universe. In this electrochemical organ, consciousness arises in a hitherto unknown way, which can perceive, feel, think and react, and which is periodically switched off and on again. Compared with these complex phenomena, the nature of the basic electrochemical process that occurs when neurons in the gray matter of the brain die under severe oxygen and glucose deprivation is relatively simple and, in effect, no different from the dying process in other cells of the body under the same conditions. Nevertheless, in the brain gray matter this electrochemical process has some interesting features that could result from the special structures that neurons have for processing information. These peculiarities include, for example, that this electrochemical process does not proceed slowly and steadily to completion as in other body cells, but abruptly reaches 90% within a few seconds about 1–5 min after the onset of severe energy deficiency, then apparently pauses for a while, and finally proceeds to completion unless the supply of oxygen and glucose resumes in time and recovery takes place. Another peculiarity is that it typically propagates slowly at a rate of 2–9 mm/min through the tissue in the form of a giant wave (Woitzik et al., 2013; Kaufmann et al., 2017; Milakara et al., 2017). Moreover, a similar giant wave can also occur in almost normal brain gray matter for largely unexplained reasons spontaneously. Under this condition, the event is only of short duration and causes comparatively mild neurological deficits. These mild deficits are referred to in neurology as migraine aura and usually disappear again without any long-term sequelae (Dreier and Reiffurth, 2015). Because neurons die faster than other body cells, it has been hypothesized that this giant wave could facilitate cell death (Gill et al., 1992; Mies et al., 1993; Busch et al., 1996; Takano et al., 1996; Dreier et al., 2007). However, definitive proof of this has yet to be provided and it is important that the wave is typically reversible at first (Luckl et al., 2018; Dreier et al., 2022). With a grain of salt (see below), the continuum from initially transient, reversible giant waves to the terminal wave can be experimentally triggered by flooding the tissue with increasing concentrations of the Na^+^/K^+^-ATPase inhibitor ouabain (Balestrino et al., 1999; Jarvis et al., 2001; Major et al., 2017). The neurons can remain in the wave state for some time without immediately dying. However, if this lasts for too long, they will die even though there is no deficiency of oxygen and glucose (Bignami and Palladini, 1966; Cornog et al., 1967; Lees and Leong, 1996). Conversely, the same giant wave can also be triggered when the Na^+^/K^+^-ATPase can no longer be activated due to a lack of oxygen, glucose and ultimately ATP (Jarvis et al., 2001; Somjen, 2001). However, if oxygen and glucose are reintroduced into the neural tissue shortly after the onset of the giant wave, ATP production starts up again, the Na^+^/K^+^-ATPase can thus be activated and the neurons survive (Ayad et al., 1994; Nozari et al., 2010; Luckl et al., 2018). The Brazilian physiologist Aristides Leão was the first to propose that this giant wave, now termed spreading depolarization, occurs in both migraine aura and cerebral circulatory arrest and is “*of the same nature*” in both conditions. He published the relevant papers on this translational hypothesis as early as 1945 and 1947 on the basis of animal observations, having described traces of the underlying phenomenon and its normal neurovascular response in animal experiments already in 1944 (Leão, 1944a, b, 1947; Leão and Morison, 1945). In particular, the experiments he describes in his 1947 paper are of refreshing intellectual clarity, as relevant today as they were more than 70 years ago, and a must read for anyone scientifically or clinically concerned with migraine aura and stroke. Specifically, Leão and Morison wrote in their 1945 paper (Leão and Morison, 1945): “Much has been written about vascular phenomena both in clinical epilepsy and the presumably related condition of migraine. The latter disease with the … slow march of scotomata in the visual or somatic sensory sphere is suggestively similar to the experimental phenomenon [the spreading depression] here described.” Leão (1947) wrote: “The results seem to indicate that in the spreading depression of activity, a change of the same nature as one resulting from prolonged interruption of the circulation, occurs in the cerebral cortex. The electrical sign of this change is the negative voltage variation.”

More than 70 years later, we have reached the point where numerous studies including five meta-analyses have confirmed the expected epidemiologic association between migraine with aura and ischemic stroke based on Leão’s original hypothesis (Etminan et al., 2005; Schurks et al., 2009; Spector et al., 2010; Hu et al., 2017; Mahmoud et al., 2018; Oie et al., 2020). The relative risk of ischemic stroke is doubled in people with migraine with aura compared with those with neither migraine with aura nor migraine without aura. In contrast, it is uncertain whether the risk of ischemic stroke is altered in migraine patients without aura (Oie et al., 2020), which is one of the arguments that migraine with aura and migraine without aura share the same headache type but generally do not share the spreading-depolarization process. Regarding the pro and con arguments in this controversy, we would like to refer to the following more comprehensive account (Dreier and Reiffurth, 2015). Most importantly, the entire spreading-depolarization continuum from short duration, to intermediate duration, to terminal waves has now been clearly demonstrated electrocorticographically in patients in all its facets and peculiarities during symptoms of migraine aura (Major et al., 2020), alternating with electrographic seizures [=ictal epileptic events (IEE)] during status epilepticus (Fabricius et al., 2008; Dreier et al., 2012; Revankar et al., 2017), in ischemic stroke (Dohmen et al., 2008; Woitzik et al., 2013; Schumm et al., 2021; Sueiras et al., 2021), in traumatic brain injury (TBI) (Strong et al., 2002; Fabricius et al., 2006; Hartings et al., 2011b), in aneurysmal subarachnoid hemorrhage (aSAH) and delayed cerebral ischemia (DCI) (Dreier et al., 2006, 2009; Bosche et al., 2010; Oliveira-Ferreira et al., 2010; Hartings et al., 2017b; Luckl et al., 2018; Sugimoto et al., 2018), including both delayed transient ischemic attacks (TIA) (Dreier et al., 2022) and delayed ischemic stroke (Luckl et al., 2018), during spontaneous intracerebral hemorrhage (Fabricius et al., 2006; Helbok et al., 2017), during subdural hematoma (Mohammad et al., 2020), during the development of brain death (Carlson et al., 2019; Dreier et al., 2019, 2022) and during the dying process from circulatory arrest (Dreier et al., 2018b, 2022). In a prospective, observational, multicenter cohort study in 138 TBI patients, the occurrence of spreading depolarization clusters was independently associated with poor outcome (Hartings et al., 2020). In DISCHARGE-1, a recent prospective, observational, multicenter, cohort, diagnostic phase III trial in 180 aSAH patients, spreading depolarization variables were included in each multiple regression model for longitudinal neuroimaging-proven early, delayed, and total brain damage, outcome at 7 months, and patient death (Dreier et al., 2022). These statistical results strongly suggest that spreading depolarizations are an independent biomarker of progressive brain injury, which is not astonishing given that spreading depolarization experimentally represents the injury potential of the brain’s gray matter (Major et al., 2020).

Surprisingly, it was not realized during several decades that there is no contradiction between the so-called vascular hypothesis of migraine by Harold Wolff and the neuronal hypothesis of Aristides Leão (see above) (Leão and Morison, 1945; Dreier et al., 2007; Dreier and Reiffurth, 2015). Wolff’s vascular hypothesis posited that migraine aura arises from intracranial vasoconstriction and migraine headache from extracranial vasodilation (Blau, 2004). It is generally assumed today that extracranial vasodilation is neither necessary nor sufficient for migraine headache (Charles and Baca, 2013; Pietrobon and Moskowitz, 2014). However, with respect to migraine aura, this traditional controversy between the vascular and neuronal theory dissolved when it became clear that epipial, i.e., abluminal, application of the vasoconstrictor polypeptide endothelin-1 is currently the most potent trigger of spreading depolarization in rodents *in vivo* and that endothelin-1 has this effect because of its vasoconstrictor properties, causing an imbalance between energy supply and demand of neurons (Dreier et al., 2002a). These findings have contributed significantly to the hypothesis that endothelial dysfunction could be one of the different etiologies leading to spreading depolarization and migraine aura in certain circumstances (Kleeberg et al., 2004; Dreier et al., 2007; Ducros, 2012; Dreier and Reiffurth, 2015; Liman et al., 2015; Oliveira-Ferreira et al., 2020; Paolucci et al., 2021). In recent clinical studies, intravenous infusion of endothelin-1 induced aura symptoms neither in healthy volunteers nor in patients with a history of migraine aura (Hougaard et al., 2020a, b). However, these clinical findings do not in any way contradict the assumption that endothelin-1 could be involved in the development of migraine auras, because endothelin-1 does not cross the blood–brain barrier and becomes a vasodilator when applied intraluminally instead of abluminally, since it causes the release of nitric oxide (NO) under this condition (Kobari et al., 1994a, b). In addition to ischemia, of course, there are countless other more or less harmful triggers for spreading depolarization. Thus, the fact that some migraine auras have a vascular etiology does not mean that all migraine auras have a vascular etiology (van den Maagdenberg et al., 2004; Leo et al., 2011; Brennan et al., 2013; Dreier and Reiffurth, 2015; Jansen et al., 2020). It has been particularly well established clinically and in animal experiments for familial hemiplegic migraine (FHM) types 1 and 3 as examples of primary neuronal disorders (Vahedi et al., 2000; Ducros et al., 2001; van den Maagdenberg et al., 2004; Dichgans et al., 2005; Jansen et al., 2020) and for FHM type 2 as an example of a primary astrocytic disorder (De Fusco et al., 2003; Jurkat-Rott et al., 2004; Dreier et al., 2005; Leo et al., 2011; Reiffurth et al., 2020; Smith et al., 2020; Parker et al., 2021) that not only primary vascular but also primary neuronal and primary astrocytic dysfunctions can lead to spreading depolarization. Indeed, it was already known before the discovery of FHM mutations that primary astrocytic dysfunction is a potent trigger of spreading depolarization (Largo et al., 1996b).

For many decades, spreading depolarization was considered by leading neurologists as a purely animal and experimental phenomenon, whose transferability to humans fell into the realm of “neuromythology” (Blau, 1992, 2004). Only a few have at least assumed that it could occur in migraine aura, but at the same time have often blanked out the fact that the occurrence of spreading depolarization in migraine aura is only the tip of the iceberg and spreading depolarizations have infinitely more implications in neurology. Ultimately, Leão was correct in highlighting the importance of spreading depolarization for both human migraine aura and human cerebral circulatory disorders based on his animal experiments (Leão and Morison, 1945; Leão, 1947). And, in retrospect, it is quite incomprehensible why this prime mechanism of acute cerebral injury in the brain’s gray matter was virtually always ignored in the preclinical preparation of the myriad, consistently negative clinical neuroprotection trials (Ginsberg, 2008). We now know that spreading depolarization ends up being the phenomenon that can be reliably reproduced across all species higher than mollusks (Spong et al., 2017; Robertson et al., 2020), and in all models with preserved cytoarchitecture (Kunkler et al., 2004; Pomper et al., 2006), and plays a key role in the mammalian brain, including the human brain, as a universal reference state in the cascades of acute neuronal injury and damage development. Once again, the dictum of Max Planck seems to prove true: “*A new scientific truth does not triumph by convincing its opponents and making them see the light, but rather because its opponents eventually die and a new generation grows up that is familiar with it*” (Planck, 1968). Many minds have contributed to the recognition of the importance of spreading depolarizations to neurology, most notably the Co-Operative Studies on Brain Injury Depolarizations (COSBID^1^), founded in 2003, which were based on a paper by Strong et al. (2002) who showed that spreading depressions of cortical activity can be recorded using subdural electrode strips in TBI patients. A particularly valuable source of inspiration for understanding the underlying mechanisms of spreading depolarization in physiological and energy-depleted tissues remains George Somjen’s book, Ions in the Brain (Somjen, 2004a).

## The Spreading-Depolarization Continuum

**FIGURE 1:**
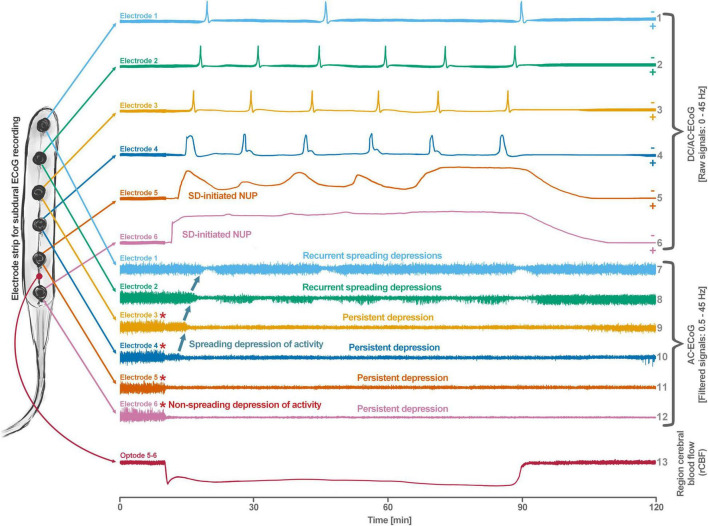
Schematic representation of the spreading depolarization (SD) continuum associated with different spontaneous brain activity changes after middle cerebral artery occlusion (MCAO). Traces 1–6 from top to bottom give the direct current (DC)/alternating current (AC)-electrocorticography (ECoG) recordings (band-pass: 0–45 Hz) where spreading depolarizations are recorded as large negative DC shifts. The ECoG traces are oriented with negativity upward and positivity downward according to the electroencephalography (EEG) convention. The following 6 traces (7–12) show the high frequency band (AC-ECoG, band-pass: 0.5–45 Hz) were changes in the spontaneous brain activity can be assessed. The last trace (13) represents the regional cerebral blood flow (rCBF) recorded with an optode for laser-Doppler flowmetry located between electrodes 5 and 6. From these recordings, we can retrieve both temporal information (*x*-axis: time) and spatial information [*y*-axis: the electrode strip is composed of 6 electrodes overlying brain cortex from the ischemic center (electrode 6) to the adequately perfused periphery (electrode 1)]. Along the *x*-axis, the first prominent event is the drop in rCBF as a result of MCAO (trace 13). A few seconds after this focal drop in rCBF, the first signs of non-spreading activity depression begin at electrodes 6–3, with the non-spreading depression being most pronounced in the ischemic center below electrode 6 (red asterisks at AC-ECoG traces 12–9). Approximately 1 min after the drop in rCBF, the first spreading depolarization begins in the ischemic center at electrode 6 and spreads concentrically from electrode 6 to electrode 1 (first negative DC shift in the DC/AC-ECoG traces 6–1). Importantly, the first spreading depolarization begins long before cell death develops, which is ultimately a consequence of toxic changes in the intraneuronal environment due to persistent spreading depolarization. Thus, the first spreading depolarization initiates a high-amplitude negative ultraslow potential (NUP) in the DC/AC-ECoG traces of electrodes 6 and 5 (traces 6 and 5), defining the later ischemic core region. In the AC-ECoG traces of electrodes 4–1, the first spreading depolarization induces spreading depression of activity (dark green arrows between traces 10–7). Note that spreading depolarization-induced spreading depression at electrode 3 (trace 9) similar to that at electrode 4 (trace 10) results in persistent activity depression, although electrode 3, unlike electrode 4, lies outside the actual ischemic penumbra (Oliveira-Ferreira et al., 2010). Spreading depolarizations in persistently depressed tissue are called isoelectric spreading depolarizations (traces 5–3 and 11–9) (Hartings et al., 2011a; Dreier et al., 2017). The first spreading depolarization at electrodes 6 and 5 cannot induce spreading depression of activity because the activity has already been suppressed by non-spreading depression of activity. Not all spreading depolarizations reach tissue remote from the ischemic center. In this example, electrode 1 only recorded three of the six spreading depolarizations. The occluded MCA is reopened after 90 min immediately followed by reperfusion (trace 13). Although necrosis develops in the ischemic core region if reperfusion occurs later than 15–20 min after the onset of ischemia (Luckl et al., 2018), the DC potential often still shows some recovery from negativity if reperfusion occurs after 90 min. Later in the course, there may even be a transient return to extremely low-amplitude spontaneous activity in the region of the ischemic core.

Spreading depolarization is observed as a negative direct current (DC) shift using electrocorticography (ECoG) (Dreier, 2011; Herreras and Makarova, 2020). The spreading-depolarization continuum describes the spectrum from transient waves with negative DC shifts of intermediate to short duration in less ischemic or adequately supplied tissue, to terminal waves in severely ischemic tissue characterized by long-lasting DC shifts and transition of the neurons from the state of acute injury to cell death (Dreier and Reiffurth, 2015; Hartings et al., 2017a). The concept of the spreading-depolarization continuum is important to understand why spreading depolarization is a prime pathophysiological process in acute neurology. The continuum is particularly easy to trace in focal cerebral ischemia secondary to proximal cerebral artery occlusion. For example, in Figure 1, the spreading depolarization continuum is shown in progressively shorter negative DC shifts along an imaginary path from the ischemic core across the penumbra to normally perfused tissue after middle cerebral artery occlusion (MCAO) (Koroleva and Bures, 1996; Dijkhuizen et al., 1999; Nallet et al., 1999). Thus, the development of cell damage in focal cerebral ischemia is characterized by wave-like exacerbations alternating with partial recoveries (Dreier et al., 2018a). The simplified and frequently used conceptual model postulates the existence of an ischemic core in which neurons die more or less immediately. In reality, however, energetic failure and spreading depolarization initiating the neuronal damage occur along a dynamic continuum in space and time (del Zoppo et al., 2011). Importantly, in focal cerebral ischemia, at least 15 min must elapse during which neurons are in the state of spreading depolarization before the first neurons in the ischemic core begin to die (Heiss and Rosner, 1983; Memezawa et al., 1992; Shen et al., 2005; Pignataro et al., 2007; Nozari et al., 2010; Luckl et al., 2018). This means that when the animals are sacrificed 72 h later, no necrotic neurons are found in the ischemic core if the ischemic core was reperfused within 15 min, even though perfusion was very low and neurons were persistently in the state of spreading depolarization during the ischemic period (Luckl et al., 2018). Around the persistently depolarized ischemic core, there is a spatially distinct region of ischemic penumbra with functionally challenged neurons that survive longer than those in the ischemic core (Hossmann, 1996). However, recurrent spreading depolarizations in the penumbra may result in increased metabolic demand, additional regional cerebral blood flow (rCBF) reduction and delayed spatial expansion of the irreversibly damaged zone that typically follows an onion-skin-like growth pattern (Gyngell et al., 1995; Busch et al., 1996; Takano et al., 1996; Dreier et al., 2007). The mechanism that ultimately leads to neuronal death can vary. In general, an extreme lack of energy causes necrotic cell death with a delay of minutes up to tens of minutes, while a milder lack of energy is more likely to result in apoptotic cell death with a delay of hours up to several days. Similar to practically all relevant mechanisms of stroke, it also applies to necrosis and apoptosis that they are not strictly separable processes, but are on a continuum, partially sharing common pathways. Furthermore, non-apoptotic regulated cell death may also contribute to the final tissue damage in stroke (Tang et al., 2019). The cell death continuum becomes apparent when cell death is followed from the ischemic core into the penumbra (Charriaut-Marlangue et al., 1996). The words ‘ischemic core’ and ‘penumbra’ thus represent gross simplifications that squeeze the high spatial and temporal complexity and variability of stroke development into a handy concept that is suitable for many practical applications in the clinic, but has unfortunately led to the misperception in scientific analysis of stroke that the pathophysiological processes in the core of ischemia are fundamentally different from the processes in the penumbra.

Only if spreading depolarization outlasts a threshold duration, the so-called commitment point, neurons will die (Somjen, 2004b). The commitment point is not a universal value but is modified by additional factors. First of all, it varies between different types of neurons involved (Heiss and Rosner, 1983; Pulsinelli, 1985). Moreover, it depends on age, local temperature, prior injury, preconditioning and the level of the remaining perfusion (Steen et al., 1979; Dreier et al., 2013b). For example, when cerebral circulation ceases completely, as after a complete cardiac arrest, and normal body temperature prevails, the commitment point shifts closer to the onset of spreading depolarization and is usually reached in less than 10 min (Steen et al., 1979; Ayad et al., 1994; Somjen, 2004b). Thus, dogs could survive only 8–9 min of complete global ischemia with return of normal neurological functions, but rCBF above 0% and below 10% of control values already prolonged the commitment point to 10–12 min, underscoring the importance of chest compressions in cardiac arrest to increase the chance of successful resuscitation (Steen et al., 1979).

By definition, a terminal spreading depolarization has two components. On the one hand, there is the initial, still reversible spreading depolarization component and, on the other hand, a late potential component, which is termed negative ultraslow potential (NUP) (Figure 1) (Oliveira-Ferreira et al., 2010; Drenckhahn et al., 2012; Hartings et al., 2017b; Dreier et al., 2018b, 2019; Luckl et al., 2018; Carlson et al., 2019). The NUP of a terminal spreading depolarization is thus experimentally defined by three typical properties: (i) that it is preceded by the initial spreading-depolarization component, (ii) that the ion shifts and cell edema do not fully recover during this extremely long DC negativity, and (iii) the death of neurons (Dreier, 2011; Dreier et al., 2013a; Luckl et al., 2018). One difficulty with this operational definition of the NUP is that the precise distinction between a NUP and a prolonged spreading depolarization that has not yet reached the commitment point and has not yet resulted in cell death cannot be made on the basis of electrophysiological criteria alone. A more detailed discussion of this can be found in the following account (Dreier et al., 2019). In human recordings with platinum/iridium electrodes, there is the additional special problem that part of the NUP is due to the interference of the electrode material with factors such as pO_2_ and pH, which may also change during ischemia in the subdural compartment where the electrodes are located (Major et al., 2021). More suitable electrode material will hopefully enable us in the near future to record the spreading depolarization-initiated NUP in humans without these superimposed interferences (Masvidal-Codina et al., 2018; Hartings, 2019). Another limitation is that the initial spreading depolarization component of terminal depolarization does not seem to be obligatory in all circumstances; it appears that terminal depolarization may also start directly with the NUP in the form of a simultaneous depolarization under certain conditions, e.g., astrocytic pre-damage (Menyhart et al., 2021).

## The Continuum of Hemodynamic Responses to Spreading Depolarization

**FIGURE 2:**
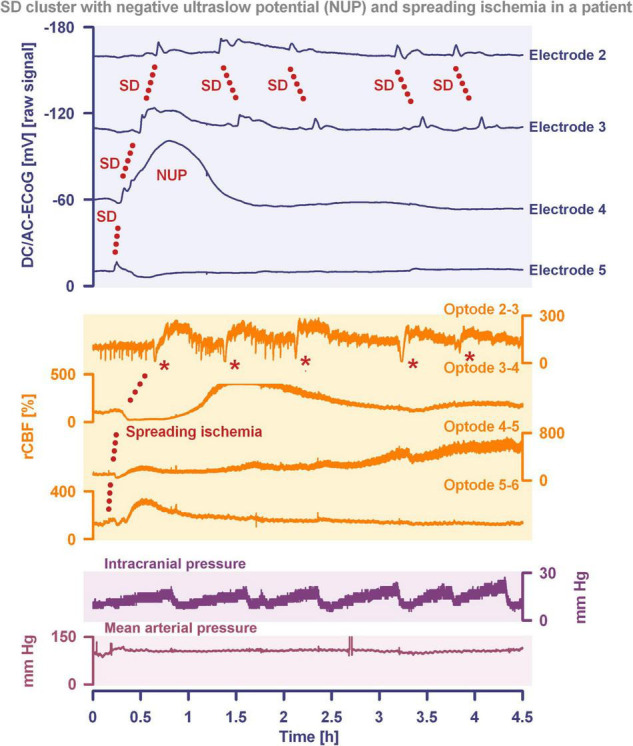
Spreading depolarization inducing a spreading ischemia and a NUP. In total, this 44-year old, previously healthy woman had 174 spreading depolarizations and died due to progressive brain infarctions on day 13 (Luckl et al., 2018). The spreading depolarizations shown in the figure were recorded on day 9 after the initial hemorrhage. Traces 1–4 from top to bottom give the DC/AC-ECoG recordings (band-pass: 0–45 Hz). Spreading depolarization is observed as a negative DC shift propagating across the cortex from electrode 5 to electrode 2. At electrode 4, the spreading depolarization initiates a NUP, the electrocorticographic correlate of infarction (Luckl et al., 2018). At electrodes 2 and 3, several shorter-lasting spreading depolarizations accompany this. Traces 5–8 give regional cerebral blood flow (rCBF) recorded with four optodes between the electrodes. At the optode between electrodes 3 and 4, the spreading depolarization induced the characteristic long-lasting drop in rCBF typical of spreading ischemia. The duration of the NUP at electrode 4 correlates well with the duration of the spreading ischemia because the decrease in perfusion and energy supply limit Na^+^/K^+^-ATPase activity and prolong the neuronal depolarization (Dreier et al., 1998; Dreier, 2011; Major et al., 2017). In contrast, only short-lasting spreading depolarization-induced initial hypoperfusions followed by hyperemias are seen at optode 2–3 (compare red asterisks). Animal experiments suggested that, if sufficiently prolonged, spreading ischemia causes necrosis (Dreier et al., 2000). Accordingly, the patient developed a new scattered infarct at electrodes 4–6 between days 8 and 12. After the spreading ischemia shown in the figure, subsequent ones also involved (opto-) electrodes 4–6. Trace 9 gives the intracranial pressure recorded using an extraventricular drainage catheter and trace 10 mean arterial pressure recorded using a catheter in the radial artery. The marked fluctuations in intracranial pressure typically result from manual opening and closing of the drainage catheter. The time is given in hours (h).

Another important continuum is the continuum of hemodynamic responses to spreading depolarization. Spreading depolarization induces tone alterations in resistance vessels, causing either predominant hyperperfusion followed by a mild oligemia (physiological hemodynamic response) in healthy tissue (Van Harreveld and Ochs, 1957; Lauritzen, 1994); or severe and prolonged initial hypoperfusion (inverse hemodynamic response = spreading ischemia) where the neurovascular unit is disturbed (Dreier et al., 1998; Dreier, 2011). Importantly, spreading depolarization can thus also be the cause of ischemia in brain tissue that was not yet ischemic at the onset of spreading depolarization (Figure 2). A necessary condition to diagnose spreading depolarization-induced spreading ischemia is that the negative DC shift of the associated spreading depolarization is prolonged as a result of the secondary mismatch between energy supply and demand (Dreier et al., 2002b; Dreier, 2011). Spreading ischemia is often followed by some recovery of rCBF up to marked hyperemia.

The phenomenon of spreading depolarization-induced spreading ischemia and inverse neurovascular coupling was discovered in a rat model, in which artificial cerebrospinal fluid (ACSF) with an elevated K^+^ concentration ([K^+^]_ACSF_) in combination with either the nitric oxide synthase (NOS) inhibitor *N*^G^-nitro-L-arginine (L-NNA) or the NO scavenger hemoglobin were topically applied to the cerebral cortex (Dreier et al., 1998, 2001, 2002b; Major et al., 2017; Sugimoto et al., 2018). Conceptually, this was a model mimicking the situation after aSAH. Similar conditions are likely responsible for spreading ischemia after arterial occlusion (Shin et al., 2006; Strong et al., 2007; Bere et al., 2014) as focal ischemia leads to a baseline elevation of the extracellular K^+^ concentration ([K^+^]_o_) before inducing spreading depolarization (Nedergaard and Hansen, 1993; Muller and Somjen, 2000; Dreier et al., 2002a) and molecular oxygen is required for NO synthesis (Jiang et al., 2001; Uetsuka et al., 2002). Ischemia-induced baseline elevation of [K^+^]_o_ in the penumbra presumably results from activation of neuronal ATP-sensitive and G protein–dependent Ca^2+^-sensitive K^+^ channels and impaired Na^+^/K^+^-ATPase function (Erdemli et al., 1998; Muller and Somjen, 2000; Revah et al., 2016). While the normal spreading depolarization-induced spreading hyperemia is assumed to be beneficial (Oliveira-Ferreira et al., 2010), spreading depolarization-induced spreading ischemia alone – i.e., without preceding ischemia – was sufficient to lead to widespread cortical necrosis (Dreier et al., 2000). The full continuum from the normal to the inverse hemodynamic response to spreading depolarization was not only measured in animals but also in patients with aSAH (Figure 2), TBI and malignant hemispheric stroke (Dreier et al., 2009; Dreier, 2011; Woitzik et al., 2013; Hinzman et al., 2014). Especially in early and delayed infarcts after aSAH, measurements suggest that spreading depolarization-induced spreading ischemia plays a dominant role in infarct development (Luckl et al., 2018; Dreier et al., 2022). Mechanistically, it is interesting to note that inverse hemodynamic responses are not limited to spreading depolarization, but also occur in response to electrographic seizures (Winkler et al., 2012) or functional activation (Koide et al., 2012; Balbi et al., 2017) after aSAH, with rCBF drops under these conditions being smaller than observed in full-blown spreading depolarization-induced spreading ischemia. These forms of dysregulatory inverse neurovascular coupling must be distinguished from physiological forms of regulatory inverse neurovascular coupling. For example, salt loading in rats physiologically leads to activation of hypothalamic magnocellular vasopressin neurons along with local vasoconstriction of pial and supraoptic nucleus parenchymal arterioles (Roy et al., 2021). Such genuine examples of inverse neurovascular responses should not be confused with responses that only appear to be inverse, but in fact correspond to normal neurovascular coupling and occur as a remote effect of local neuronal activation. For example, unilateral stimulation and thus activation of the locus coeruleus area leads to ipsilateral inhibition of the cingulate cortex (Dillier et al., 1978). In terms of normal neurovascular coupling, there is then a decrease in local rCBF in the cingulate cortex in response to the local neuronal deactivation (de la Torre, 1976).

## Ion and Transmitter Changes During Spreading Depolarization and Seizures

It is assumed that spreading depolarization is a primarily neuronal summary process during which the transmembrane gradients of virtually all the small molecules we can measure change. That is, spreading depolarization consists of the interplay of millions of individual processes, which of course involve not only neurons but the entire neurovascular unit and microglial cells. For a few of these changes, more or less precise quantifications exist. For example, the extracellular Na^+^ concentration ([Na^+^]_o_) drops from ∼147 to ∼60 mM and the intraneuronal Na^+^ concentration ([Na^+^]_i_) increases from ∼10 to ∼36 mM (Kraig and Nicholson, 1978; Hansen and Zeuthen, 1981; Kager et al., 2002; Windmuller et al., 2005; Hubel and Ullah, 2016; Gerkau et al., 2017). The extracellular Ca^2+^ concentration ([Ca^2+^]_o_) decreases from ∼1.3 mM to ∼80 μM and the intraneuronal Ca^2+^ concentration ([Ca^2+^]_i_) increases from ∼60 nM to at least ∼25 μM (Hansen and Zeuthen, 1981; Windmuller et al., 2005; Marino et al., 2007; Dietz et al., 2008; Hubel and Ullah, 2016; Revah et al., 2016). In comparison, the changes in these concentrations during an epileptic seizure are much smaller: [Na^+^]_o_ drops from ∼150 to ∼140 mM, [Na^+^]_i_ increases from ∼10 to ∼16 mM (Lux et al., 1986; Kager et al., 2002), [Ca^2+^]_o_ drops from ∼1.3 to ∼0.9 mM and [Ca^2+^]_i_ increases from ∼60 to ∼130 nM (Wadman et al., 1985; Pisani et al., 2004; Kovacs et al., 2005; Marino et al., 2007).

In parallel with the Na^+^ and Ca^2+^ influx, there is a K^+^ efflux from the neurons during spreading depolarization. That is, [K^+^]_o_ increases from ∼3 to ∼50 mM during spreading depolarization, whereas the intracellular K^+^ concentration ([K^+^]_i_) decreases from ∼135 to ∼112 mM (Vyskocil et al., 1972; Kraig and Nicholson, 1978; Hansen et al., 1980; Hansen and Zeuthen, 1981; Perez-Pinzon et al., 1995; Kager et al., 2002; Windmuller et al., 2005; Hubel and Ullah, 2016). In comparison, the changes during an epileptic seizure are again much smaller: [K^+^]_o_ increases from ∼3 to ∼12 mM and [K^+^]_i_ decreases from ∼135 to ∼127 mM (Heinemann and Lux, 1977; Dreier and Heinemann, 1991; Kager et al., 2002). The situation is similar for the increase in neurotransmitters. For example, while the extracellular glutamate concentration increases from ∼2 to ∼4 μM during an epileptic seizure (Li et al., 2018), it increases up to ∼100 μM during spreading depolarization (Zhou et al., 2013).

Importantly, despite these mild but significant metabolic disturbances during a seizure and the severe metabolic disturbances during spreading depolarization, neither a seizure nor a spreading depolarization immediately kills neurons if they are of short duration and occur in tissues that are adequately supplied with oxidative substrates and not exposed to toxins (Nedergaard and Hansen, 1988; Zachrisson et al., 2000; Besse et al., 2020). However, when they occur over a longer period of time, both seizures and spreading depolarizations can cause cellular damage. Only a few papers have directly compared the cell-damaging potency of electrographic seizures and spreading depolarizations. For example, in a study of photothrombosis in the rat, no increase in lesion volume or neuronal damage was observed in the early time window by inducing electrographic seizures with 4-aminopyridine under urethane anesthesia, whereas ketamine/xylazine anesthesia reduced the number of spontaneous spreading depolarizations and resulted in a smaller lesion volume and less neuronal damage, although 4-aminopyridine-induced electrographic seizures were concomitantly enhanced because the antiepileptic effect of ketamine is less than that of urethane (Schoknecht et al., 2021). In a recent cohort study of patients with TBI, the occurrence of spreading depolarization clusters was significantly associated with worse patient outcomes in a multivariate ordinal regression model adjusting for baseline prognostic variables (Hartings et al., 2020). In the same cohort, electrographic seizures were significantly associated with severity and number of spreading depolarizations (Foreman et al., 2022). However, in contrast to clustered spreading depolarizations, electrographic seizures detected on either ECoG or scalp electroencephalography (EEG) showed no independent association with functional outcome at 6 months after controlling for known prognostic covariates and the presence of spreading depolarizations. Thus, consistent with the much greater changes in ion gradients and neurotransmitters during spreading depolarization, these studies suggest that spreading depolarizations are fundamentally more dangerous than epileptic seizures, although, on the other hand, a single short-lasting spreading depolarization is more benign than, for example, generalized status epilepticus. A caveat, however, is that it has not been well enough studied how often spreading depolarizations occur superimposed on status epilepticus. In many status epilepticus models, spreading depolarizations typically co-occur with electrographic seizures (Koroleva and Bures, 1983; Mody et al., 1987; Hablitz and Heinemann, 1989; Avoli et al., 1991; Tamim et al., 2021).

## The Basal Mechanism of Spreading Depolarization

Fortunately, to understand the basal process underlying spreading depolarization, we do not need to know all the many small molecules whose transmembrane gradients change during spreading depolarization. Initially, we do not even need to know what a neuron is, but a basic knowledge of thermodynamics and electrochemistry might be helpful. For those who have not studied thermodynamics and electrochemistry in a while, there are textbooks or websites for a refresher [e.g., Map: Chemistry – The Central Science (Brown et al.) (Anon, 2021). Chapter 19: Chemical Thermodynamics and Chapter 20: Electrochemistry^2^.]. With knowledge on entropy and the second law of thermodynamics, entropy changes during chemical reactions, oxidation states and redox reactions, cell potentials, Gibbs free energy, batteries, fuel cells, and electrolysis, it is possible to move on to the so called Gibbs–Donnan equilibrium. For example, the Gibbs–Donnan equilibrium is well explained in the following articles (Sarkar et al., 2010; Sperelakis, 2011). Anyone who understands what a Gibbs–Donnan equilibrium is has a basic understanding of why spreading depolarizations occur in the central nervous system (CNS) of all properly investigated insects and vertebrates under pathological conditions. In the following, we would like to briefly recapitulate the basics of this.

The physiological state of mammalian body cells is electrochemically characterized by two opposing Gibbs–Donnan effects. The first Gibbs–Donnan effect arises from the high concentration of intracellular macromolecules. These are predominantly proteins that are negatively charged both in physiological and even highly pathological pH ranges. Importantly, the cell membrane is impermeable to these macromolecules, i.e., they are trapped inside the cell. If this first Gibbs–Donnan effect is not compensated by another force, it imposes an unequal distribution of permeant charged ions such as Na^+^, K^+^, and Cl^–^ ions on either side of the semipermeable cellular membrane. When an equilibrium is reached, namely the so-called Gibbs–Donnan equilibrium, (1) each bulk solution is electrically neutral on either side of the cellular membrane, (2) the product of diffusible ions on one side of the membrane is equal to the product of diffusible ions on the other side of the membrane, (3) the electrochemical gradients due to the unequal distribution of charged ions lead to a transmembrane potential difference, which can be calculated using the Nernst equation and is slightly negative on the inner side of the membrane, (4) an osmotic diffusion gradient is created attracting water into the intracellular compartment, and (5) the cells swell produced by water uptake. Although it cannot be completely ruled out that neurons can survive the complete establishment of a Gibbs–Donnan equilibrium for a very short time, we know of no experimental case in which this has been demonstrated. If, on the other hand, the cerebral cortex is freshly dead after circulatory arrest, the ion changes measured, without any exception to this rule being known to us, are consistent either with a Gibbs–Donnan equilibrium or with a state in which cell lysis has already begun as a result of cellular swelling (Hansen and Zeuthen, 1981).

Thus, in order for Gibbs–Donnan equilibrium not to occur, an opposing force is needed in body cells to balance the first Gibbs–Donnan effect. This opposing force is generated by the so-called second Gibbs–Donnan effect, for which not non-diffusible macromolecules in the intracellular space, but quasi non-diffusible Na^+^ ions in the extracellular space are responsible (Sperelakis, 2011; Dreier et al., 2013a). These generate and maintain the osmotic countergradient for water movement. The result is a steady state characterized by isoosmolarity between intracellular and extracellular space, so that there is no net transmembrane water movement. The entrapment of Na^+^ ions in the extracellular space is based on two mechanisms. First, the cell membrane is nearly impermeable to Na^+^ ions under physiological conditions. Second, the ATP-powered Na^+^ pump (Na^+^/K^+^-ATPase) transports three Na^+^ ions out of the cell in exchange for two K^+^ ions (Skou and Esmann, 1992). This means that excess Na^+^ ions are immediately transported back out of the cell. In summary, the two opposing Gibbs–Donnan effects, due to entrapment of non-diffusible macromolecules in the intracellular space and entrapment of quasi non-diffusible Na^+^ ions in the extracellular space, produce the double Gibbs–Donnan steady state that characterizes the physiological state of body cells.

As long as the brain of a born human being is “healthy,” the neurons are without any exception in the double Gibbs–Donnan steady state both while awake and asleep and throughout life. In any case, no exceptions to this rule have become known so far. However, excellently reproducible ion measurements and measurements of the volumes of the intracellular and extracellular compartments by many different research groups over several decades, discussed in more detail above and below, have shown that under pathological conditions two types of deflection are possible, in which the brain gray matter shifts within a few seconds from the double Gibbs–Donnan steady state toward the Gibbs–Donnan equilibrium. In both cases, the deflection initially stalls in a new metastable state without reaching the Gibbs–Donnan equilibrium. However, these two metastable states are much more unstable than the physiological double Gibbs–Donnan steady state, which in turn is less stable than the Gibbs–Donnan equilibrium. The first type of deflection is small, reaching a metastable state still close to the physiological double Gibbs–Donnan steady state, and is usually referred to as an electrographic epileptic seizure. The second type of deflection is large, close to Gibbs–Donnan equilibrium, and referred to as spreading depolarization (Dreier et al., 2013a).

A complete Gibbs–Donnan equilibrium becomes established in body cells when the Na^+^/K^+^-ATPase stops working. As already mentioned, this can happen when the pump is directly inhibited or because it lacks fuel, i.e., the necessary ATP. It is important to note that the pathological Gibbs–Donnan equilibrium does not require metabolic energy in the form of ATP for its formation; it evolves passively because its formation releases Gibbs free energy that drives the process. This is unlike the physiological double Gibbs–Donnan steady state. The physiological double Gibbs–Donnan steady state requires active ion transport by membrane pumps and the use of metabolic energy in the form of ATP to establish the large transmembrane electrochemical gradients of Na^+^, K^+^, Ca^2+^, etc. Thus, in the physiological double Gibbs–Donnan steady state, the entropy of the system is kept artificially low by the use of ATP, and in this way electrochemical energy is stored in the system, which is then available to do work. From this electrochemical energy store, the neurons take the necessary energy to be able to send out their signals, the so-called action potentials. However, it is important to understand that although the membrane potential changes dramatically for a millisecond during an action potential, as some Na^+^ and K^+^ ions cross the membrane, the transmembrane concentration gradients between the bulk solutions of the intracellular and extracellular space remain virtually unchanged, implying that the cell remains in the physiological double Gibbs–Donnan steady state. This means that the energy store loses almost no energy during one action potential. Otherwise, the system would not be able to function. This should be imagined like a flashlight that can be turned on and off repeatedly without discharging the entire battery in the short term.

A characteristic of the physiological double Gibbs–Donnan steady state is a strongly negative resting membrane potential across the cell membrane. However, the membrane potential at the pathological Gibbs–Donnan equilibrium is also not zero, but is normally between 0 and −20 mV (Sperelakis, 2011). In the pathological Gibbs–Donnan equilibrium, all permeant ions are in equilibrium across the cell membrane and the equilibrium potentials for all permeant ions such as K^+^ and Cl^–^ are of the same magnitude and polarity, whereas this is not the case in the physiological double Gibbs–Donnan steady state. The membrane potential at the pathological Gibbs–Donnan equilibrium arises even if the cell membrane has the same permeability or conductivity for all small ions, whereas the normal resting membrane potential of the physiological double Gibbs–Donnan steady state requires different conductivities for, e.g., Na^+^ and K^+^ ions, namely a significantly higher conductivity for K^+^ ions than for Na^+^ ions. In the pathological Gibbs–Donnan equilibrium, the osmolarity of the cell increases relative to the osmolarity of the extracellular space because more Na^+^ ions flow into the cell than K^+^ ions flow out due to Gibbs–Donnan forces imposed by the large, negatively charged, intracellular macromolecules. In order for the bulk solutions on either side of the cell membrane to remain electrically neutral, Cl^–^ ions must also enter the cell in addition to the Na^+^ ions, but there are slightly less Cl^–^ ions flowing in than Na^+^ ions, since there are also some K^+^ ions flowing out. In Gibbs–Donnan equilibrium, the product of the small diffusible positive and negative ions on one side of the membrane is then equal to the product of the small diffusible positive and negative ions on the other side of the membrane, even though the sum of the small diffusible positive and negative ions in the intracellular space is greater than the sum of the small diffusible positive and negative ions in the extracellular space, so that the osmolarity of the intracellular space is greater than the osmolarity of the extracellular space. Driven by the difference in osmolarity between the extracellular and intracellular space, water follows Na^+^ and Cl^–^ ions into the cell, resulting in what is known as cytotoxic edema (Dreier et al., 2013a, 2018a).

Neurons, unlike astrocytes, are normally relatively resistant to purely extracellular osmolarity changes because they lack regular water channels (Aitken et al., 1998; Andrew et al., 2007). On this basis, it is postulated that only membrane processes during spreading depolarization, i.e., the opening of specific channels during spreading depolarization, which are not yet clearly elucidated, allow water to follow the osmolarity gradient that builds up between the intraneuronal and extracellular space. The influx of water then leads to a marked swelling of neuronal dendrites and somas within a few seconds during spreading depolarization (Kirov et al., 2020). For example, candidate neuronal channels that could be used as water channels during spreading depolarization include membrane transporters (Steffensen et al., 2015). However, the exact mechanism is unclear, and in these considerations it is important to keep in mind that regardless of which pore the water enters through during spreading depolarization, ATP cannot be required for it. In other words: Water transport mechanisms that require energy cannot be involved in the water influx into neurons during spreading depolarization.

There are also no satisfactory explanations yet for the abrupt non-specific increase in Na^+^/K^+^ conductance that mediates Na^+^ influx and K^+^ efflux during spreading depolarization (Czeh et al., 1993). Thus, the forces, namely the Gibbs–Donnan forces, that drive spreading depolarization are clear, but the exact pathways by which the large amounts of Na^+^, K^+^, Ca^2+^, Cl^–^, and water move across the cell membrane from one compartment to another are not.

However, since these facts are somewhat counterintuitive, they are sometimes misrepresented and it is then claimed, for example, that K^+^ efflux and Na^+^ influx during spreading depolarization are the same (Hellas and Andrew, 2022) or intra- and extracellular ion concentrations equilibrate during spreading depolarization. However, as previously pointed out by Somjen (2001), the reasoning error underlying such interpretations is mainly based on the failure to take into account the volume ratios and volume changes between the intracellular and extracellular space. Conversely, the conclusion that more Na^+^ ions enter neurons than K^+^ ions leave them during spreading depolarization (Somjen, 2001; Dreier et al., 2018a) is not based on “dogma” in “textbooks” (Hellas and Andrew, 2022) but results from quantitative measurements of [Na^+^]_o_, [K^+^]_o_, [Cl^–^]_o_ (Vyskocil et al., 1972; Kraig and Nicholson, 1978; Hansen et al., 1980; Hansen and Zeuthen, 1981; Lehmenkuhler, 1990; Perez-Pinzon et al., 1995; Somjen, 2001; Windmuller et al., 2005), [Na^+^]_i_ (Gerkau et al., 2017), and the extracellular (Vorisek and Sykova, 1997; Mazel et al., 2002; Windmuller et al., 2005) and intracellular volume changes (Takano et al., 2007; Murphy et al., 2008; Kirov et al., 2020), which have been performed and reproduced by many different research groups in different decades. Thus, [Na^+^]_o_ drops from ∼147 to ∼60 mM, that is, by ∼87 mM, but [K^+^]_o_ increases from 3 to ∼50 mM and thus by only ∼47 mM. Correspondingly, [Cl^–^]_o_ drops from ∼135 mM to at least ∼95 mM and thus by approximately 40 mM (Kraig and Nicholson, 1978; Hansen and Zeuthen, 1981; Windmuller et al., 2005). How many times more Na^+^ ions flow from the extracellular space into the intracellular space than K^+^ ions flow out can be estimated from the measured data. As mentioned above, it is important to consider the volume ratios and volume changes of the intracellular and extracellular space. While the percentage of extracellular space is 18–22% of the total volume under physiological conditions, it declines to 5–9% during spreading depolarization (Hansen and Olsen, 1980; Jing et al., 1994; Perez-Pinzon et al., 1995; Mazel et al., 2002; Windmuller et al., 2005). In other words, the extracellular space declines by ∼70%. If this 70% shrinkage of the extracellular space in favor of the intracellular space were due to a pure water shift and no ions crossed the cellular membranes, then [Na^+^]_o_ should increase from ∼147 to ∼490 mM and [K^+^]_o_ from ∼3 to ∼10 mM. However, [Na^+^]_o_ actually decreases from ∼147 to ∼60 mM, while [K^+^]_o_ increases from ∼3 to ∼50 mM. Assuming [Na^+^]_i_ at ∼10 mM and [K^+^]_i_ at ∼135 mM under physiological conditions (Kager et al., 2002) and taking into account the measured extracellular ion changes as well as intra- and extracellular volume changes during spreading depolarization, [Na^+^]_i_ should increase from ∼10 to ∼36 mM and [K^+^]_i_ should decrease from ∼135 to ∼112 mM during spreading depolarization. Here, most of the [K^+^]_i_ decrease results from the dilution effect due to water uptake, whereas the net loss of K^+^ ions from the intracellular space is small. This is in stark contrast to the [Na^+^]_i_ increase, which is entirely due to the net gain in Na^+^ ions caused by the Na^+^ influx, while the dilution effect due to water uptake counteracts the increase in [Na^+^]_i_. From the concentration and volume changes it can then be calculated that about 11 times more Na^+^ ions enter the intracellular space than K^+^ ions leave during spreading depolarization. Indeed, neuronal [Na^+^]_i_ of ∼24 and ∼30 mM measured during spreading depolarization *in vivo* and *in vitro* are quite close to the estimated value (Gerkau et al., 2017). The small discrepancy between measured and estimated values should take into account that, in addition to ion shifts between the extracellular space and the cytoplasm, there are very likely also ion shifts between the cytoplasm and intracellular organelles (Hernansanz-Agustin et al., 2020). For quantitative measurements of [Na^+^]_i_ *in vivo*, it must additionally be considered that these optical measurements are made relatively superficially and the ion changes tend to be smaller there than deep in the cortex. Quantitative [K^+^]_i_ measurements do not yet exist for spreading depolarization because the available K^+^ dyes do not have sufficient selectivity.

Overall, the extracellular ion measurements are in good agreement with the theory in textbooks of chemistry and biochemistry. Importantly, the ion measurements suggest not only that more Na^+^ ions flow into the cells than K^+^ ions flow out, but also that the extracellular space loses ∼80 mosmol/l in favor of the intracellular space during this process. The decrease in extracellular osmolarity and the smaller increase in intracellular osmolarity relative to the larger intracellular volume are also in good agreement with the textbook theory. Since not only the distributions of the major small ions Na^+^, K^+^, and Cl^–^ across the cell membrane change during spreading depolarization, but also the concentrations of countless other molecules, and since not only neurons but also other cell types, especially astrocytes (Somjen, 2001), are involved, the complexity is certainly higher than portrayed here, but it is nevertheless not likely that the basic properties of the process change just because even more molecules and cell types are involved. Nevertheless, it would certainly be useful to develop methods to directly measure intracellular and extracellular osmolarity changes during spreading depolarization to test these conclusions.

Fortunately, then, it is not necessary to overturn the fundamentals of electrochemistry to understand spreading depolarization. In other words, during spreading depolarization the system comes very close to the pathological Gibbs–Donnan equilibrium without fully reaching it, making spreading depolarization an important metastable reference state of general interest (Kraig and Nicholson, 1978; Kager et al., 2002; Dreier et al., 2013a; Hubel and Ullah, 2016). When cells die, the pathological Gibbs–Donnan equilibrium is finally reached unless the cell membrane lyses first. Na^+^ ions probably play a less passive role in all these processes than has long been assumed, because there is increasing evidence that Na^+^ ions, like Ca^2+^ ions (Wolf et al., 2017), have second-messenger functions. For example, Na^+^ ions are now thought to be directly involved in the control of mitochondrial ATP production (Hernansanz-Agustin et al., 2020; Geisberger et al., 2021).

In case the reader is now still not familiar with the principle of spreading depolarization, the authors would like to suggest a practical exercise: the reader may connect the positive terminal of her/his car battery to the negative terminal and then try to drive around with the car. With a grain of salt, the shorted car battery is a car battery in the state of spreading depolarization and the inability of the car to do any work in this situation – i.e., drive around with the battery shorted – is a car in spreading depression of activity.

In conclusion, neuronal cytotoxic edema is the morphological correlate of the near-complete neuronal battery breakdown called spreading depolarization, or conversely, spreading depolarization is the electrophysiological correlate of the initial, still reversible phase of neuronal cytotoxic edema (Van Harreveld, 1957, 1958; Hubel and Ullah, 2016; Dreier et al., 2018a; Kirov et al., 2020). Cytotoxic edema and spreading depolarization are thus different modalities of the same process (Major et al., 2020). Accordingly, the swelling of the neuronal somas and the so-called beading of the dendrites as a result of the water influx can be detected in two-photon laser scanning microscopy simultaneously with the negative shift of the DC potential (Andrew et al., 2007; Takano et al., 2007; Murphy et al., 2008; Risher et al., 2010, 2011; Steffensen et al., 2015). These facts are important for the understanding of water diffusion restrictions in the gray matter, which can be detected by magnetic resonance imaging (MRI) (Dreier and Reiffurth, 2017). Thus, the beaded morphology during spreading depolarization allows the neurons to enclose a larger volume of water within a constant surface area (Budde and Frank, 2010). In normal dendrites, water mobility is strongly constrained by the cell membrane perpendicular to the major axis, whereas water molecules diffusing along the major axis of the dendrites encounter few obstacles on the time scale of diffusion-weighted (DW) MRI. However, if water influx during spreading depolarization leads to dendritic beading – i.e., alternating between severe dendritic constrictions and balloon-like dilations – water can no longer diffuse along the major dendrite axis because water jams at the dendritic constrictions. This intracellular diffusion restriction during spreading depolarization can be imaged with DW-MRI (Busch et al., 1995, 1996; Gyngell et al., 1995; Hoehn-Berlage et al., 1995; Hossmann, 1996; Takano et al., 1996; Dreier and Reiffurth, 2017). The technology has now advanced to the point where it is experimentally possible to map the propagation path of spreading depolarization in the cortex and subcortical gray matter in high resolution and three dimensions using DW-MRI (Cain et al., 2017). The vascular neurologist who looks at DW-MRIs of her/his stroke patients should be aware that reversible diffusion restriction in the gray matter (Fiehler et al., 2004) results from spreading depolarization and persistent diffusion restriction from the transition to a NUP (Figures 1, 2). However, in the case of a locally very short-lasting spreading depolarization, e.g., in the context of a normal migraine aura, the depolarized wavefront would be expected to be so narrow that it should be very difficult to detect diffusion restriction in this region with standard clinical MRI scanners.

## The Overarching Theme of Spreading Depolarization

The overarching theme inherent in all spreading depolarizations comprises the near-complete breakdown of the transmembrane neuronal ion gradients that cause the water influx and neuronal swelling, the marked neuronal and astroglial depolarization, the changes in holding current and input resistance of patch-clamped neurons, intrinsic optical signal (IOS) changes, the abrupt release of neurotransmitters, including both excitatory neurotransmitters such as glutamate and inhibitory neurotransmitters such as GABA, and the fall in tissue ATP (Mies and Paschen, 1984; Somjen, 2001; Molchanova et al., 2004; Aiba and Shuttleworth, 2014; Dreier and Reiffurth, 2015). However, the changes in spontaneous neuronal activity are not part of this list because spreading depolarization may very well occur in tissues without any spontaneous activity, and the changes in spontaneous brain activity around the occurrence of spreading depolarization may exhibit large temporal and spatial variations, as explained below. The hemodynamic response to spreading depolarization is also not included in this list of common characteristics of all spreading depolarizations because spreading depolarization can also occur in brain slices that do not have an intact blood circulation, and the changes in rCBF around spreading depolarization *in vivo* can also have large temporal and spatial variations as explained above.

The Gibbs free energy released when the physiological double Gibbs–Donnan steady state transitions to the epileptic seizure state, to the state of spreading depolarization, and finally to death – that is, the Gibbs–Donnan equilibrium – can be estimated. Using simple models of neuropil, changes in cation concentrations and electric field alone resulted in a Gibbs free energy release of ∼3 J/l per tissue volume when the network entered the epileptic seizure state and of 19–22 J/l per tissue volume when it entered spreading depolarization (Dreier et al., 2013a). The subsequent transition from the spreading depolarization state to cell death resulted in an additional small free energy release of 2.5 J/l. The Gibbs free energy released is converted to heat. Based on the above estimates, tissue temperature should increase by 5 mK during spreading depolarization. This is only slightly smaller than the measured temperature increase between 5 and 30 mK in isolated bullfrog and toad retinas (Tasaki and Byrne, 1991). These estimates once again emphasize that spreading depolarization/cytotoxic edema is a thermodynamic reference state of living neurons near cell death, at which ∼90% of the Gibbs free energy contained in the ion gradients has been lost (“’free energy starving”’) (Dreier and Reiffurth, 2015; Hubel and Ullah, 2016). The hypothesis that spreading depolarization is the mechanism of the neuronal cytotoxic edema is owed to Anthonie van Harreveld, who coined it before the term cytotoxic edema even existed (Van Harreveld, 1957, 1958; Dreier et al., 2018a).

Basically, spreading depolarizations can be measured via all million-fold concentration changes of molecules in the intracellular and extracellular space that are not freely diffusible and have a non-uniform distribution across the cell membrane, as well as via many other physicochemical properties (Major et al., 2020). Thus, to detect spreading depolarizations in principle, and especially their local duration, it probably makes relatively little difference whether the DC potential, [Na^+^]_i_, [Na^+^]_o_, [Ca^2+^]_i_, [Ca^2+^]_o_, [K^+^]_i_, [K^+^]_o_, [Cl^–^]_i_, [Cl^–^]_o_, pH_i_, pH_o_, DW-MRI, cellular swelling, extracellular shrinkage, and so on are recorded. Whenever a new measurement method is found, it is naturally sold as better and more ingenious than the previous one. Fortunately, over time, a more realistic view develops and it is possible to move on to discussing pros and cons in a factual manner. For example, questions of interest include (1) whether the method is suitable for measuring spreading depolarizations in patients, (2) whether it is suitable for non-invasive measurement in animals and/or patients, (3) whether it allows imaging, (4) whether it has higher spatial and/or temporal resolution than previous methods, (5) whether it combines well with other recording methods and/or (6) allows more accurate quantification of individual processes nested in the overall process of spreading depolarization. In addition, it is also interesting when the new method provides new mechanistic insights, for example, regarding the mechanism underlying the large changes of membrane permeability, the mechanism of propagation, the brain activity changes, the continuum of hemodynamic responses or the edema formation. The latter not only includes the cytotoxic edema (Dreier et al., 2018a). Thus, with a delay, spreading depolarizations also induce the ionic edema (Mestre et al., 2020) and subsequently blood–brain barrier disruption and vasogenic edema (Gursoy-Ozdemir et al., 2004; Sadeghian et al., 2018).

Even though spreading depolarization primarily begins in neurons (Peters et al., 2003; Chuquet et al., 2007), a separate review can in principle be written about each cell type involved. For example, astrocytes are of paramount importance for the orderly process of spreading depolarization. To give a few examples, astrocyte-directed inactivation of connexin 43 decreased astrocytic gap junctional communication and increased tissue susceptibility to spreading depolarization and the propagation speed of spreading depolarization (Theis et al., 2003). Selective intoxication of astrocytes caused spreading depolarizations that initiated a shallow NUP as neurons began to die (Largo et al., 1996b). Glia dysfunction may accelerate neuronal death under ischemic conditions (Largo et al., 1996a; Kimelberg, 2005). Accordingly, impaired astrocyte function can abolish the typical slow spread of spreading depolarization in the ischemic zone and cause the commitment point to occur earlier (Menyhart et al., 2021).

Electrographic seizures can usually be easily distinguished from spreading depolarizations. The reader will find details on this in the following articles (Dreier et al., 2017; Revankar et al., 2017). Hybrid phenomena between spreading depolarizations and epileptic activity are further discussed below. Of note, although there are many models that lead to both epileptic seizures and spreading depolarizations (Koroleva and Bures, 1983; Mody et al., 1987; Hablitz and Heinemann, 1989; Avoli et al., 1991; Dreier et al., 2013a; Tamim et al., 2021), there are also important differences, and the type of increased excitability that leads to seizures is by no means the same as the one resulting in spreading depolarizations. For example, GABA_A_ agonists are first-line drugs for the treatment of epileptic seizures and limit the propagation speed of spreading depolarization (Aiba et al., 2012). On the other hand, GABA is released during spreading depolarization (Molchanova et al., 2004) and may contribute to neuronal swelling through the influx of Cl^–^ ions (Allen et al., 2004; Dreier and Reiffurth, 2015). In addition, it was recently shown that a mutation of the Na^+^ channel NaV1.1, which in humans leads to FHM type 3, one of the Mendelian model diseases of spreading depolarization, is associated with impaired inactivation of the channel (Jansen et al., 2020). The interesting point is that this excitatory Na^+^ channel is mainly expressed by GABAergic interneurons and the impaired inactivation should lead to increased inhibition in the network. Nevertheless, the mutation increases the propensity to spreading depolarization. In contrast, mutations leading to a loss of function instead of a gain of function of the same channel cause epilepsy syndromes such as Dravet syndrome, a pharmacoresistant developmental and epileptic encephalopathy, and genetic epilepsy with febrile seizures plus (Escayg et al., 2000; Claes et al., 2001). Another important example of divergence between epileptic excitability and excitability leading to spreading depolarization is epileptogenesis, the long-term, plastic process resulting in epilepsy. Thus, the propensity to spreading depolarization appears to decrease during epileptogenesis, although the propensity to spontaneous seizures increases (Koroleva et al., 1993; Tomkins et al., 2007; Maslarova et al., 2011).

## There Are No Disease-Specific Spreading Depolarizations

Spreading depolarization represents the injury potential of the brain’s gray matter associated with the neuronal cytotoxic edema (Major et al., 2020). Although the neural network need not tip into ruin when spreading depolarization occurs, spreading depolarization nevertheless typically occurs when the neural network tips into ruin, and second, the local duration of the spreading depolarization state indicates whether or not the neural network irreversibly perishes (Dreier et al., 2017). Of course, there are exceptions to all rules. For example, evidence has recently been found that selective ischemic death of Purkinje cells does not require the occurrence of spreading depolarization (Oliveira-Ferreira et al., 2020). Furthermore, the fact that locally long-lasting spreading depolarizations in regions of impaired metabolism lead to neuronal damage does not preclude that locally short-lasting spreading depolarizations in regions of unimpaired metabolism surrounding an emerging lesion might have beneficial adaptive properties and signaling functions (Dreier, 2011; Shuttleworth et al., 2019). There is a need for research in both directions, as these questions are relevant to which neuroprotective interventions might be useful. In all of this, it is important to note that spreading depolarizations can occur in a plethora of clinical conditions, but there are no disease-specific spreading depolarizations, just as epileptic seizures can occur in a plethora of clinical conditions that are sometimes more and sometimes less benign, but there are no disease-specific epileptic seizures. This means, for example, that there are no specific migraine spreading depolarizations. Thus, a spreading depolarization that has migrated into the normally perfused surroundings of an ischemic zone will hardly differ there from a spreading depolarization as it occurs in the context of a migraine with aura. It is therefore advisable to detach a moment from the clinical zeal for classification and to embrace scientific abstraction. As explained above, the overarching theme is that both epileptic seizures and spreading depolarizations are network accidents that inevitably result from the way neural networks are configured. When certain neural network safety systems are overridden, the network must respond with epileptic seizures and/or spreading depolarizations because it has no other choice. Epileptic seizures and spreading depolarizations do not result from network decisions, but the network is forced to undergo these partial breakdowns of small magnitude (epileptic seizure) or massive magnitude (spreading depolarization) by the second law of thermodynamics (Dreier et al., 2013a; Hubel and Ullah, 2016). The second law of thermodynamics establishes the concept of entropy as a physical property of a thermodynamic system. During an epileptic seizure or a spreading depolarization, the entropy of the system abruptly increases, which, as explained above, is associated with the release of Gibbs free energy, which in turn is converted into heat. Thus, an epileptic seizure or a spreading depolarization proceed voluntarily (passively) and can only be terminated by the expenditure of ATP (actively), especially for the Na^+^/K^+^-ATPase. The ATPase actively lowers the entropy of the system again and in this way replenishes the electrochemical energy store of the cells.

What should not remain unmentioned in this context is the approach, which we interpret either as a little ruse or as a genuine misunderstanding, how even today it is often justified that spreading depolarizations in migraine are always harmless and have not much to do with spreading depolarizations in stroke. This consists of calling spreading depolarizations in sublethal doses spreading depression and in lethal doses anoxic depolarization. Then a spreading depression is always harmless by definition, because at the moment when the neurons unfortunately died, it was not a spreading depression but an anoxic depolarization. But if they survive, then by definition it was not an anoxic depolarization but a spreading depression. This is like writing one pharmacopeia for harmless substances and another pharmacopeia for hazardous substances and listing the same substances in both, but with different names, and justifying this by saying that, on the one hand, each of the substances in sublethal doses is not lethal and therefore harmless, and, on the other hand, each substance in lethal doses is of a different nature because it is lethal. How and when this *secundum quid et simpliciter* type of fallacy developed is difficult to trace. The fallacy dissolves when Paracelsus’ concept comes into play that the dose makes the poison. His full quotation reads: “Poison is in everything, and no thing is without poison. The dosage makes it either a poison or a remedy.” In the context of the summary process of spreading depolarization, which is ultimately a concert of thousands or millions of more or less parallel processes, it might even be worthwhile to think more deeply about the whole meaning of the quote, including the part about the potential healing effects of possible poisons, as already discussed above (Dreier, 2011; Shuttleworth et al., 2019). Moreover, it should be added that the term dose in the context of spreading depolarization means the cumulative dose of the potentially noxious changes, such as the 500–1,000-fold increase in [Ca^2+^]_i_ (Dietz et al., 2008, 2009; Revah et al., 2016), as a function of exposure time, i.e., as a function of the local duration of spreading depolarization (Dreier et al., 2017). As a rule of thumb, one locally long-lasting spreading depolarization is more dangerous than 100 locally short-lasting spreading depolarizations (Figures 1, 2). In this context, it should also be briefly mentioned that a spreading depolarization is certainly not a homogeneous process, but has different phases, such as a wave front and a sustained phase, which often has a saddle-shaped or even more complex forms (Somjen, 2001). The changing mechanisms of these phases may well be important for the toxicity of a given spreading depolarization (Herreras and Somjen, 1993; Aiba and Shuttleworth, 2012; Reinhart and Shuttleworth, 2018; Mei et al., 2020).

Unfortunately, when the intermediate range of the spreading-depolarization continuum was increasingly encountered in rodent MCAO experiments since the early 1980s, the rigid separation between anoxic depolarization and spreading depression was not immediately abolished. Instead, a third type of wave was postulated, which was termed peri-infarct depolarization. However, we believe that this term also conveys a misleading concept, because when the first spreading depolarizations occur during MCAO, no infarct is yet present and, accordingly, these spreading depolarizations cannot be called peri-infarct depolarizations. In this context, it is important that the pathologist defines an infarct and an ischemic stroke, respectively, by the ischemic necrosis of neurons and not by decreases in cerebral perfusion, tissue partial pressure of oxygen (p_ti_O_2_), tissue glucose or tissue ATP, or by an initially reversible state of depolarized neurons. That we have used the terms “spreading depression,” “peri-infarct depolarization,” and “anoxic depolarization” in this twisted manner in the past, when knowledge was less, is hopefully forgivable (Dreier et al., 1998) and should not diminish the value of the earlier publications that used this nomenclature, but to continue to use this twisted nomenclature today is problematic because it cements a concept that is ultimately misleading. A continuum (cf. Figures 1, 2) should not be cut into rigid parts to pretend that they are unrelated. By analogy, light waves, for example, also differ greatly in their properties as a function of wavelength and yet can only be interpreted and quantified if it is understood that they are part of the light wave continuum. What makes all this particularly difficult is the fact that the term ‘spreading depression’ is still needed for what it really denotes, namely the spreading depression of spontaneous activity caused by spreading depolarization. So the point is not to stop using the term spreading depression, but to use it correctly. In the last section, we will explain how spreading depolarization and spreading depression differ from each other.

## The Role of the Na^+^/K^+^-Atpase in Spreading Depolarization

Sufficient activity of the Na^+^/K^+^ pump protects against spreading depolarization because, as explained above, the pump is responsible for maintaining the second Gibbs–Donnan effect by trapping Na^+^ ions in the extracellular space, which counteracts the first Gibbs–Donnan effect due to the high concentration of negatively charged intracellular macromolecules. For the same reason, tissue recovery from spreading depolarization is not possible, if Na^+^/K^+^ pump activity is insufficient. All three α isoforms of the Na^+^/K^+^ pump presumably antagonize the spreading depolarization process. Their roles, however, vary, as they differ in their cellular and subcellular localizations, kinetic properties and affinities to Na^+^, K^+^ and ATP (O’Brien et al., 1994). Thus, the housekeeping α_1_ isoform is found on all vertebrate cells, is very sensitive to both K^+^ and Na^+^, has the greatest turnover and shows an intermediate dependence on the membrane potential. Accordingly, this isoform works at optimum rates under physiological conditions but seems to respond less well to demands (Crambert et al., 2000; Reiffurth et al., 2020). By contrast, the α_2_ isoform, which is located on glial cells but not on neurons in the adult brain, is ideally suited to oppose acute rises in [K^+^]_o_ because of its low affinity to K^+^. This low affinity permits the enzyme to rapidly adapt its turnover over a wide range of [K^+^]_o_ (Grisar et al., 1980; Walz and Hertz, 1982). In addition, α_2_ activity rises in response to the membrane depolarizing effect of increasing [K^+^]_o_ (Crambert et al., 2000; Horisberger and Kharoubi-Hess, 2002), which applies in particular when the α_2_ isoform is linked with the β_2_ subunit (Stoica et al., 2017). Accordingly, the K^+^ threshold for spreading depolarization was exclusively lower in α_2_ isoform-deficient mice in contrast to α_1_ or α_3_ isoform-deficient mice (Reiffurth et al., 2020) and mutations in the ATP1A2 gene, encoding the α_2_ isoform, cause FHM type 2, a rare Mendelian model disease of spreading depolarization (De Fusco et al., 2003; Jurkat-Rott et al., 2004; Dreier et al., 2005; Leo et al., 2011; Unekawa et al., 2018). In contrast to the α_2_ isoform, the α_3_ isoform is found on neurons. This isoform is characterized by a low Na^+^ affinity which allows it to respond particularly well to the marked rise in [Na^+^]_i_ during spreading depolarization, suggesting its involvement in the neuronal recovery from spreading depolarization (Crambert et al., 2000). However, application of ouabain at 100 μM in interface slices of rodents should fully block the α_2_/α_3_ portion of the Na^+^/K^+^ pump. Yet, ouabain at 100 μM induces a prolonged but largely reversible spreading depolarization (Balestrino et al., 1999). This means that high intraneuronal Na^+^-induced activation (Skou and Esmann, 1992) of the less ouabain-sensitive α_1_ isoform alone is still sufficient to largely reestablish the ion gradients and repolarize the neurons during spreading depolarization. In contrast, complete blockade of all three isoforms by a very high dose of ouabain leads to terminal spreading depolarization, that is, the Gibbs–Donnan equilibrium and death of the cells.

An interesting detail is that prolonged exposure to slightly elevated [K^+^]_o_ levels appears to decrease the activity of certain Na^+^/K^+^-ATPases, which may explain a decrease in the K^+^ threshold for spreading depolarization with increasing duration of exposure to slightly elevated [K^+^]_o_. Thus, Major et al. (2017) measured a selective decline in α_2_/α_3_ Na^+^/K^+^-ATPase activity only 30 min after exposure to elevated [K^+^]_o_ below the K^+^ threshold of spreading depolarization. Importantly, the *ex vivo* enzyme assay was performed under optimal biochemical conditions, suggesting that the prior prolonged exposure to elevated [K^+^]_o_ *in vivo* must have caused a persistent disturbance of the pump, which was still detectable *ex vivo*. A similar decline in activity was also previously observed in cultured astrocytes within 10 min of [K^+^]_o_ elevation to a relatively high concentration of 30 mM (Hajek et al., 1996). Overall, the experimental data currently indicate that this suppression of α_2_/α_3_ enzyme activity by prolonged [K^+^]_o_ elevation results from modifications of specific active sites. This could trap the enzyme in an inactive phosphorylated state (Mishra and Delivoria-Papadopoulos, 1999).

Apart from this detail, it has been found that all Na^+^/K^+^-ATPases are strongly activated over the entire range of [K^+^]_o_ and [Na^+^]_i_ levels reached during spreading depolarization (Skou and Esmann, 1992; Crambert et al., 2000; Horisberger and Kharoubi-Hess, 2002; Larsen et al., 2014). In other words, the activability of Na^+^/K^+^-ATPases by [K^+^]_o_ and [Na^+^]_i_ is not abolished as a result of the abrupt maximal increases of [K^+^]_o_ and [Na^+^]_i_, because otherwise spreading depolarization would always be irreversible. Together with the fact that ouabain at high concentrations that inhibit all three α isoforms prevents recovery from spreading depolarization, all the known properties of the Na^+^/K^+^-ATPases (Skou and Esmann, 1992; Crambert et al., 2000; Horisberger and Kharoubi-Hess, 2002; Larsen et al., 2014) strongly support the notion that their increased activation is a necessary mechanism for recovery from spreading depolarization.

The question remains as to what structure underlies the non-specific Na^+^/K^+^ conductance that initially drives spreading depolarization (Czeh et al., 1993) and whereby this increase in permeability of the cell membrane disappears again, rather than that Na^+^/K^+^-ATPases are additionally required to terminate spreading depolarization. While we do not rule it out, we do not consider it very likely that the structure underlying the non-specific Na^+^/K^+^ conductance is the same as that responsible for recovery from spreading depolarization. That is, we do not consider it likely that the conversion of one or more Na^+^/K^+^-ATPases into channels drives spreading depolarization (Robertson et al., 2020), because it would then be difficult to explain why spreading depolarization is in principle a reversible phenomenon. Also, this hypothesis would hardly be suitable to explain why spreading depolarization with the characteristic abrupt depolarization, the abrupt near-complete breakdown of the ion gradients and the recovery occurring at the earliest after several tens of seconds has so far only been detected in the CNS, since Na^+^/K^+^-ATPases are also present in all other tissues. The α_1_ isoform prevails in all vertebrate cells, but the α_2_ and α_3_ isoforms also exist not only in the CNS (Blanco, 2005). Although the search for the non-specific Na^+^/K^+^ channels in neuronal membranes that drive spreading depolarization has been unsuccessful to date, it remains the most likely hypothesis that such channels exist (Makarova et al., 2007; Dreier et al., 2013a), that they are relatively specific to neurons, and that they are structures that are different in nature from those required to restore ion homeostasis after the onset of spreading depolarization. The caveat is added, however, that it has not yet been sufficiently investigated whether spreading depolarization is indeed so specific to the gray matter of the brain or whether similar phenomena may also occur in other tissues of the body. A first step might be to characterize the exact time course of depolarization and breakdown of ion gradients during intoxication with ascending concentrations of the Na^+^/K^+^-ATPase inhibitor ouabain using ion-sensitive microelectrodes in comparative studies between brain and other organs. Flooding of brain gray matter with increasing concentrations of ouabain results in a continuum of initially reversible, single and clustered spreading depolarizations with progressively broadened negative DC shifts starting from an elevated level of baseline [K^+^]_o_ and returning to that elevated baseline (Balestrino et al., 1999; Major et al., 2017). Eventually, a terminal spreading depolarization occurs (Jarvis et al., 2001). Overall, the changes are very similar to those produced by ischemia of various severities. Alternatively, focal ischemia could also be directly investigated in comparative studies between brain and other organs.

In such experiments it is important to understand that the postulated principal difference between neurons and other body cells is not that the other body cells do not also swell when the Na^+^/K^+^-ATPase inhibitor ouabain is administered or ischemia is induced, but that other body cells swell very slowly over many hours. For example, erythrocytes do not swell in response to ouabain in the form of sudden events as neurons in the brain gray matter do in the form of spreading depolarizations (Kowluru et al., 1989). The fundamental difference is that the Na^+^ permeability of erythrocytes remains extremely low when ouabain is administered, so that the evolution from the physiological double Gibbs–Donnan steady state to Gibbs–Donnan equilibrium is very slow. On the other hand, if Na^+^ permeability is increased artificially by toxins such as palytoxin while Na^+^/K^+^-ATPase is inhibited, the swelling process and lysis of erythrocytes can be accelerated significantly (Volpe et al., 2014).

## Brain Activity Changes in the Context of Spreading Depolarization

A fair question is then how all this translates into changes of neural function. The simplest way to observe neural function is to measure changes in spontaneous activity. Spontaneous brain activity results from the firing of cortical neurons. The firing of upstream neurons causes postsynaptic potentials in downstream neurons. These postsynaptic potentials are associated with rapid extracellular field potential changes. Synchronous activity from thousands or millions of neurons with similar spatial orientation produces fast extracellular field potential changes that are electrocorticographically recorded as brain electrical activity. As is well known, the minor breakdown, namely the epileptic seizure, involves an increase in firing rate and increased synchronization in the network, and the major breakdown, the spreading depolarization, results in loss of function, that is, silencing of spontaneous activity (Leão, 1944b). This spreading depolarization-induced spreading depression of spontaneous activity is observed as a rapidly evolving reduction in the amplitudes of spontaneous activity in the alternating current (AC) frequency band of the ECoG above 0.5 Hz and spreads along with spreading depolarization between neighboring recording sites (Figure 1). This migratory extinction of spontaneous activity was discovered by Leão (1944b) and termed spreading depression of spontaneous activity before he demonstrated the underlying process in 1947, i.e., the large negative DC shift. Leão (1947) interpreted the latter as an expression of neuronal depolarization. He based this interpretation on the similarity between the DC shift he recorded in the cerebral cortex and the asphyxia-induced DC shift recorded in the spinal cord by Van Harreveld and Hawes (1946), who previously interpreted this negative DC shift as reflecting neuronal depolarization. It is assumed that spreading depolarization initiates spreading depression because the sustained depolarization exceeds the inactivation threshold for the action potential generating channels (Kager et al., 2002). In practice, however, the observed patterns often deviate from expectations and sometimes depression begins, for example, only when the negative DC shift is already in regression, or it does not occur at all. If depression occurs, it usually lasts significantly longer than the negative DC shift, suggesting that it is maintained by other mechanisms than the depolarization block such as intracellular zinc or Ca^2+^ and/or extracellular adenosine accumulation (Lindquist and Shuttleworth, 2012; Carter et al., 2013; Sawant-Pokam et al., 2017).

Already Leão (1944b, 1947) discovered additional patterns of spontaneous activity changes that can co-occur with the large negative DC shift of spreading depolarization such as non-spreading depression of activity or epileptic field potentials that replace the spreading depression of activity. The first signs of non-spreading depression start within seconds of anoxia, focal or global circulatory arrest with an arousal reaction of fast, irregular low-voltage activity (Dreier and Reiffurth, 2015). Synchronous gamma oscillations then occur within the first 30 s which are global and highly coherent with a striking increase in anterior-posterior-directed connectivity (Borjigin et al., 2013). Isoelectricity is usually reached within 30–40 s, well before the neuronal ATP pool is depleted (Figure 1) (Hochachka et al., 1996). As opposed to spreading depolarization-induced spreading depression, non-spreading depression develops simultaneously in the whole area exposed to severe ischemia. Also of particular importance is that non-spreading depression is associated with neuronal hyperpolarization in contrast to the depolarization block that initiates spreading depression (Tanaka et al., 1997; Muller and Somjen, 2000). It is still unclear how hypoxic neurons and astrocytes are able to sense the drop in p_ti_O_2_ but several mechanisms have been proposed that may mediate non-spreading depression. These include: (i) changes in vesicular transmitter release (Katchman and Hershkowitz, 1993b; Fleidervish et al., 2001; Revah et al., 2016), (ii) activation of ATP-sensitive or G protein–dependent Ca^2+^-sensitive K^+^ channels (Erdemli et al., 1998; Muller and Somjen, 2000), (iii) release of adenosine (Fowler, 1989; Katchman and Hershkowitz, 1993a; Khazipov et al., 1993, 1995; Dzhala et al., 1999; Canals et al., 2008), (iv) acidosis (Mutch and Hansen, 1984; Taylor et al., 1996), and (v) breakdown of gamma oscillations (“interneuron energy hypothesis”) (Kann et al., 2014). In animals, non-spreading depression of activity is typically measured in the center of ischemia after MCAO or during anoxia or global circulatory arrest (Leão, 1947; Mayevsky and Chance, 1975; Luckl et al., 2018) (Figure 1). In humans, it has only been clearly demonstrated so far in the dying process during cardio circulatory arrest (Dreier et al., 2018b). Non-spreading depression of activity usually significantly precedes the first spreading depolarization in the center of ischemia. The median latency between cessation of spontaneous activity and onset of spreading depolarization was 65 s in the center of ischemia after MCAO in rats (Luckl et al., 2018) and 76 s in the dying process in humans during cardio circulatory arrest (Dreier et al., 2018b). Non-spreading depression is thought to lead to the sudden and simultaneous neurological deficits in various modalities, such as language, motor, sensory, or visual functions, that are typical of transient ischemic attacks and most types of ischemic strokes (Dreier and Reiffurth, 2015).

**FIGURE 3:**
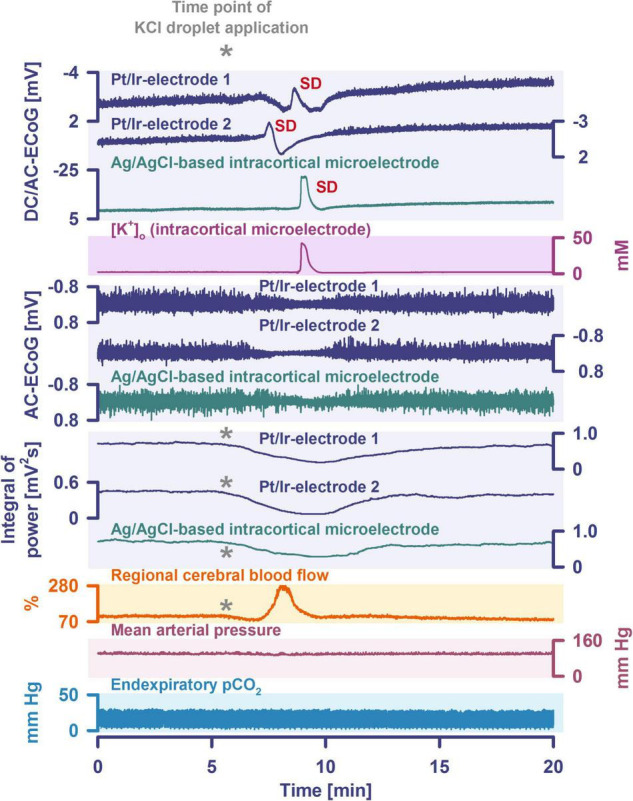
Spreading depolarization remotely triggered by a droplet of KCl (2 M) in a naïve rat *in vivo* is shown. Traces 1–3: the full-band ECoG signal [DC/AC-ECoG] contains information on both the negative DC shift that identifies spreading depolarization (Dreier, 2011) and the activity changes. Note the differences in amplitude and shape of the DC shift between the gold standard recording technique in animals, the intracortical glass-microelectrode, and the platinum/iridium (Pt/Ir) electrodes on the brain surface, gold standard in humans (Dreier et al., 2017; Major et al., 2021). Also note the spread of the spreading depolarization in rostral (Pt/Ir-electrode 2)-caudal (Pt/Ir-electrode 1) direction. Trace 4: the hallmark of spreading depolarization is the near-complete breakdown of the transcellular ion concentration gradients. Here, the large spreading depolarization-associated increase in [K^+^]_o_ is seen. Traces 5–7: the spontaneous activity is assessed in the AC-ECoG, i.e., the higher frequency band between 0.5 and 45 Hz. Traces 8–10 display the integral of power of the spontaneous activity (Dreier et al., 2006, 2017). The integral of the power of the AC-ECoG is based on a method of computing time integrals over a sliding window according to a time decay function. This mathematical procedure provides a smoothed curve easing visual assessment of changes in AC-ECoG power. The method has become standard to score depression durations accompanying spreading depolarization. Spreading depolarization shows a depressive effect on spontaneous activity (=spreading depression). However, other events can also cause depression of activity. For example, we observed here a simultaneous depression of spontaneous activity at all electrodes immediately after the rostral KCl application (cf. asterisks). A subsequent, additional spreading depolarization-induced depression is observed at the caudal Pt/Ir electrode. Trace 11: a shallow initial hypoperfusion accompanied the KCl-triggered simultaneous depression of spontaneous activity that preceded the spreading depolarization-induced hyperemia. Trace 12 shows the mean arterial pressure (measured via femoral artery catheter). Trace 13 shows the end-expiratory pCO_2_.

However, simultaneous depressions of spontaneous activity at different recording sites may precede spreading depolarization not only in the context of ischemia. For example, they can typically be detected even at some distance from a KCl droplet application to trigger spreading depolarization *in vivo*, even if no local increase in [K^+^]_o_ precedes the spreading depolarization at the affected recording site (Figure 3). The underlying mechanism of this type of simultaneous depression, however, is unknown.

Another curiosity, described as early as 1944 (Leão, 1944b) and 1953 (van Harreveld and Stamm, 1953), is that the silencing of brain activity induced by spreading depolarization changed as a result of minimal electrical stimulations. Finally, epileptic field potentials were recorded during the period that had originally seen spreading depression of activity. Van Harreveld and Stamm called this phenomenon spreading convulsion. It is characterized by epileptic field potentials superimposed on the negative DC potential. Most often these occur on the final shoulder of the negative DC potential. Spreading convulsions have been observed, e.g., in organotypic cultures (Kunkler and Kraig, 1998) but also in human brain slices and in patients (Dreier et al., 2012). Based on a discussion with our colleagues Jeff Noebels, Jed Hartings, and Bill Shuttleworth, it is probably more appropriate to use the term spreading depolarization with epileptiform activity (SDEA) instead of spreading convulsion to refer to the phenomenon, since it is not a behavioral phenomenon but an electrographic phenomenon.

**FIGURE 4:**
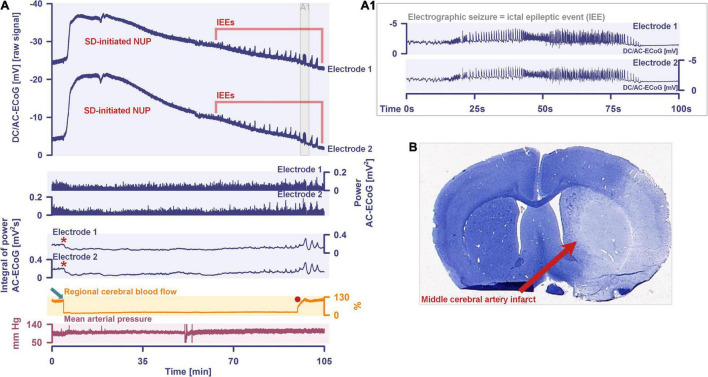
**(A)** Spreading depolarization-initiated NUP with superimposed ictal epileptic events (IEEs) after middle cerebral artery occlusion (MCAO) in a rat (Luckl et al., 2018). Traces 1 and 2 show the full-band ECoG signal [DC/AC-ECoG] which contains both the negative DC shifts that identify spreading depolarizations and the changes in spontaneous brain activity. Traces 3 and 4 only show the spontaneous activity in the higher frequency band (AC-ECoG, bandpass: 0.5–45 Hz). Traces 5 and 6 display the integral of the power of the AC-ECoG which provides a smoothed curve easing visual assessment of changes in AC-ECoG power. Trace 8: regional cerebral blood flow (rCBF) recorded with laser-Doppler flowmetry. Trace 9: mean arterial pressure measured via femoral artery catheter. MCAO leads to a sharp decrease in rCBF (dark green arrow in trace 7). Shortly after MCAO, non-spreading depression of activity occurs and is best observable in the integral of the power (red asterisks at traces 5 and 6). The drop in rCBF triggers a spreading depolarization that propagates from electrode 2–1 and initiates a NUP (traces 1 and 2). Electrographic seizures, also known as IEE, occur superimposed on the NUP. They continue and even intensify after the reperfusion (red dot at trace 7). **(A1)** Displays one of the IEEs at higher temporal resolution. **(B)** The animal was sacrificed 72 h later. The infarct in both basal ganglia and cortex is macroscopically identified as a pale area using hematoxylin staining.

**FIGURE 5:**
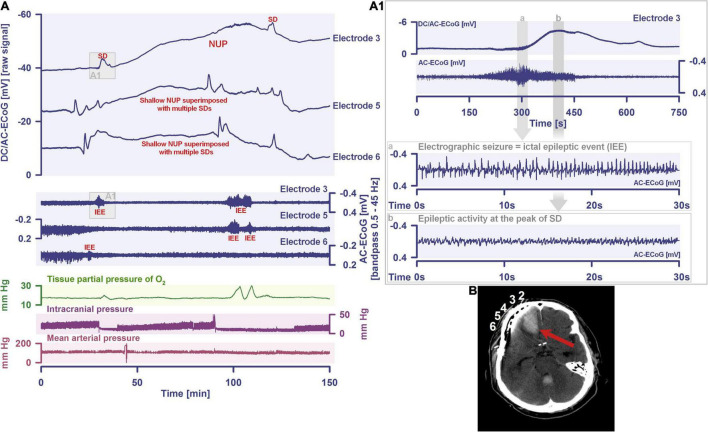
**(A)** Spreading depolarization-initiated NUP superimposed with further SDs and IEEs recorded after aneurysmal subarachnoid hemorrhage (aSAH) in a 71-year old man (Dreier et al., 2012; Winkler et al., 2012). The NUP occurred on day 10 after the initial hemorrhage. Traces 1–3 from top to bottom give the DC/AC-ECoG recordings (band-pass: 0–45 Hz). The SD-initiated NUP and the later superimposed SDs are observed as negative DC shifts (negative upward). Traces 4–6 not only show the depressive effect of the SDs on the spontaneous brain activity but also the superimposed IEEs (AC-ECoG, band-pass: 0.5–45 Hz). Note that the first IEE at electrode 3 (trace 4) is temporally associated with the onset of an SD [shown at higher temporal resolution in the insert **(A1)**]. Since the IEE is at least partially superimposed on the negative DC shift, it is a spreading convulsion according to the old nomenclature (van Harreveld and Stamm, 1953) or a spreading depolarization with epileptiform activity (SDEA) according to the new nomenclature. Trace 7 shows the tissue partial pressure of oxygen (p_ti_O_2_) measured with an intraparenchymal sensor. Trace 8 gives the intracranial pressure (ICP) measured with an extraventricular drainage (EVD) catheter. Trace 9 shows the mean arterial pressure (MAP) measured with a radial artery catheter. The IEEs are associated with hyperoxic responses (the oxygen sensor was located in the cortex between electrodes 3 and 4). **(B)** Post-operative computed tomography (CT) scan showing an intracerebral hematoma (red arrow) in the right frontal lobe of the patient and electrodes 2–6 of the subdural recording strip.

**FIGURE 6:**
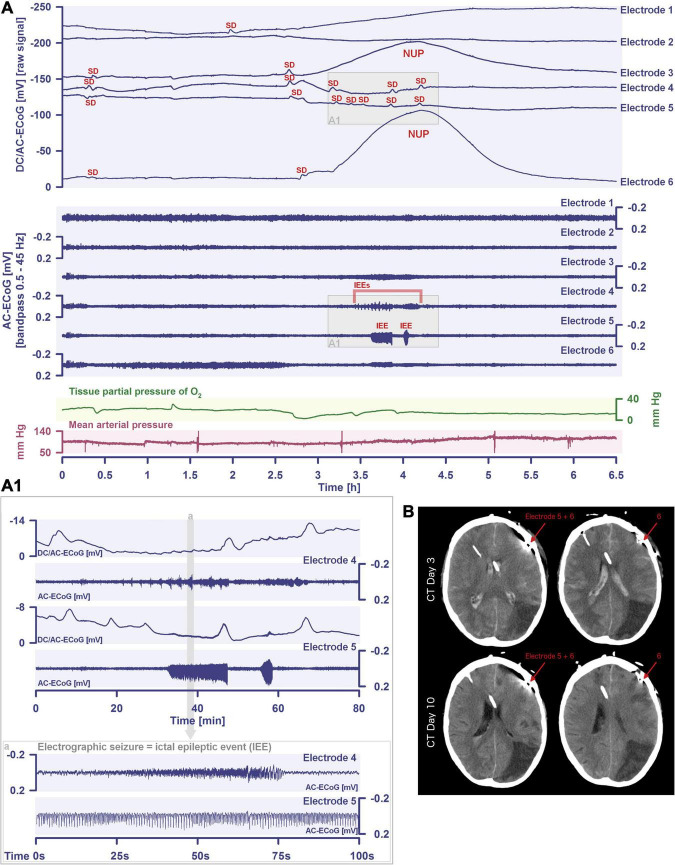
**(A)** A cluster of SDs initiating a NUP is shown that occurred in a 57-year old man after aneurysmal subarachnoid hemorrhage. The cluster was recorded with a 6-contact subdural strip for ECoG on day 7 after the initial hemorrhage. Traces 1–6 from top to bottom give the DC/AC-ECoG recordings (band-pass: 0–45 Hz). Spreading depolarizations and spreading depolarization-initiated NUP are observed as negative DC shifts (negative upward). Traces 8–12 show persistent depression of the spontaneous activity after the first spreading depolarization at electrodes 3–5 as assessed in the high frequency band (AC-ECoG, band-pass: 0.5–45 Hz). This depression is interrupted by IEEs best seen at electrodes 4 and 5 [traces 10 and 11; shown at higher temporal resolution in the insert **(A1)**]. Trace 13: tissue partial pressure of oxygen (p_ti_O_2_) measured with an intraparenchymal sensor. Trace 14: mean arterial pressure measured with a radial artery catheter. **(B)** Compared with computed tomography (CT) at day 3 after the initial hemorrhage, CT at day 10 shows new hypodensities typical of scattered delayed ischemic infarction in the cortex near the subdural electrodes, matching the spreading depolarization-induced NUP at electrode 6 on day 7. Accurate assessment is somewhat limited by the typical hyperdense streak artifacts around the electrodes.

A special case of such SDEA occurs when the electrographic seizures overlie a terminal spreading depolarization, i.e., a spreading depolarization-initiated NUP, rather than a short-lasting spreading depolarization. Examples are given in Figure 4 after MCAO in the rat and in Figures 5, 6 in aSAH patients. Such patterns can probably be explained by portions of the network jumping from the near-complete sustained depolarization of spreading depolarization to the less pronounced sustained depolarization of electrographic seizures. In animal experiments, this pattern was associated with particularly large infarcts (Luckl et al., 2018). However, this was most likely related to the fact that the associated NUPs were particularly pronounced.

On the basis of all these observations, although spreading depression is an excellent term for the spreading depression of activity, it is misleading and tantamount to semantic paraphasia to use the term spreading depression as a generic term for the underlying process of spreading depolarization. This is especially true as it has been widely reproduced that spreading depolarization can also occur in tissues without any spontaneous neuronal activity such as in normoxic and normoglycemic brain slices (Dreier et al., 2001). In such tissues we would then have to speak of ‘spreading depression in absence of spreading depression,’ if the term ‘spreading depression’ is used once as a generic term instead of the term ‘spreading depolarization’ and on the other hand denotes the spreading depression of spontaneous activity, which is clearly absurd. Instead, the COSBID group has recommended to use the term spreading depolarization as the generic term for the underlying process (Dreier et al., 2017). However, this should in no way prevent us from using the term spreading depression for the spreading depression of activity, i.e., where this term is scientifically correct. For example, spreading depolarization-induced spreading depression of activity in Figure 1 is more pronounced the farther the electrode is from the center of ischemia because the farther the electrode is from the center of ischemia, the less pronounced (or even absent) the non-spreading depression of activity. Furthermore, whereas the dying process in cardio circulatory arrest usually leads first to non-spreading depression and then to spreading depolarization, the development of brain death during continued circulation in the intensive care unit is most commonly initiated by spreading depolarization and induced spreading depression (Dreier et al., 2018b, 2019, 2022; Carlson et al., 2019).

A particular problem in migraine research is that quite a few migraine researchers are convinced that all spreading depolarizations of interest to the migraine researcher induce spreading depression, which is thought to entail the patient percept of the migraine aura (Leão and Morison, 1945), and spreading depolarizations, once accompanied by other spontaneous activity changes, no longer have anything to do with migraine and therefore are not relevant to the migraine researcher. However, this is at odds with the thin data available. Indeed, there is only a single case report in the world literature to date in which spreading depolarization-induced spreading depression was electrocorticographically recorded while the patient had symptoms of a migraine aura, i.e., the patient reported the sequential series of neurological deficits typical of migraine aura (Major et al., 2020). She even had two migraine auras in succession, during each of which spreading depolarization-induced spreading depression was recorded. However, the patient had subdural electrodes implanted only because she had suffered aSAH a few days earlier, and she had no previous history of migraine with aura or without aura. Apart from one more study investigating magnetoencephalographic fields from patients during spontaneous and induced migraine aura (Bowyer et al., 2001), the other evidence supporting that migraine aura is caused by spreading depolarization is all indirect and based on the similarity of recorded rCBF or blood oxygen level-dependent (BOLD) changes during migraine aura with the normal hemodynamic response to spreading depolarization in animal studies (Olesen et al., 1981; Lauritzen et al., 1983; Hadjikhani et al., 2001). However, not only is the evidence from these studies indirect, but the studies using the planar intracarotid ^133^Xenon method and single-photon emission computed tomography (SPECT) in patients undergoing migraine auras were additionally problematic as the migraine auras recorded in these studies cannot be referred to as normal migraine auras. Very likely, not only spreading depressions but also non-spreading depressions of spontaneous activity may have occurred in the context of spreading depolarizations in these studies, because it is assumed that the large number of recorded migraine auras in these studies resulted from the procedure of catheterizing and injecting the carotid artery, which provoked visual migraine aura in more than 50% of the patients (Lassen and Friberg, 1991). It is now widely assumed that the mechanism leading to these auras was cerebral microemboli causing brief ischemic episodes, because such microemboli are potent triggers of spreading depolarizations in animals (Nozari et al., 2010; Dreier and Reiffurth, 2015). That is, these studies were essentially a proof of Leão’s original hypothesis that the basal neural network processes during migraine aura and during disturbances of the cerebral circulation are “*of the same nature*.”

Although most spreading depolarizations that occur in association with migraine aura are unlikely to be primarily vascular in origin, the alert neurologist working in a stroke unit or as a consultant in a cardiology department where there is a high volume of interventions such as cardiac catheterization and atrial ablation will see single cases of migraine aura associated with minor, often pinpoint stroke every year if the patients are carefully interviewed and MRIs are performed (Olesen et al., 1993; Klingebiel et al., 2008; Dreier and Reiffurth, 2015; Waters et al., 2018; Altamura et al., 2021). Also, to understand why relatively small strokes can sometimes be accompanied by transient global neurological deficits (Dreier et al., 2006) or why transient focal symptoms can sometimes occur whose representational fields are not in the ischemic zone proper (Klingebiel et al., 2008), it is useful to have an understanding of the spreading-depolarization continuum, because spreading depolarizations are precisely not only local but can spread over large areas of cortex and subcortical gray matter (James et al., 1999; Cain et al., 2017; Kaufmann et al., 2017; Milakara et al., 2017). For basic researchers, the erroneous use of the term ‘spreading depression’ for the phenomenon of ‘spreading depolarization’ is particularly problematic because, often believing that the changes in spontaneous activity are irrelevant, they use inadequate filter settings for the ECoG, i.e., record only the DC potential, and all valuable information from the experiments contained in the changes in spontaneous activity is lost. This is especially relevant when the experiments are used to correlate electrophysiology and behavior.

## Discussion

Spreading depolarization is one of the most important pathophysiological processes of the CNS, with far-reaching implications for neurology, and there, not only for migraine with aura, but also for the most diverse forms of acute injury of the cerebrum and most likely also of the cerebellum, brainstem and spinal cord, and even for paroxysmal movement disorders (Lu et al., 2021). Spreading depolarization is among the most basic pathological processes in the nervous system that appeared in phylogenesis even before the development of a proper blood circulation (Spong et al., 2017). The reason why spreading depolarization must occur in insects and vertebrates under certain conditions is derived from the fundamental structure of their neural networks and, in particular, from the second law of thermodynamics. In this context, the role of certain neurotransmitters in the nervous systems of different species (Robertson et al., 2020), such as the role of glutamate, may be fundamentally different without altering the basic property of spreading depolarization, which is that the state of the neural network shifts abruptly toward the pathological Gibbs–Donnan equilibrium, but without reaching it completely unless the neurons eventually die.

For individuals suffering from migraine with aura, all this means that they should minimize their vascular risk factors, even though their absolute risk of stroke is low. On the one hand, patients with typical migraine with aura should be reassured that the constellations under which spreading depolarizations occur in migraine with aura are almost always benign, and they should not be subjected to inappropriate and potentially dangerous investigations and treatments. On the other hand, if the migraine aura occurs in an unusual constellation, the treating physician should be alert and perform further diagnostic workup, as it may conceal a stroke (Olesen et al., 1993; Klingebiel et al., 2008; Waters et al., 2018). It is also particularly important to understand that migraine is not a prerequisite for the occurrence of spreading depolarizations, and very many spreading depolarizations do not manifest clinically as migraine auras. Any stroke that affects the gray matter is presumably associated with spreading depolarizations, and almost all of us will end up with a terminal spreading depolarization regardless of whether or not we had migraine auras during our lifetime (Dreier et al., 2018b, 2019, 2022; Carlson et al., 2019). Exceptions are of course conceivable, starting with the fact that one can also die from the immediate impact of a bomb.

It may be added that the fact that spreading depolarization does not occur in highly artificial systems such as cell cultures is not an argument against a thorough investigation of this process; on the contrary, it is another argument in favor of it. Indeed, there are a number of prominent examples where the results of cell culture experiments may be consistent in themselves, but the experiments do not examine the *in vivo* state of the animal or patient that they purport to examine. For example, as explained above, spreading depolarization secondarily leads to a massive increase in the concentrations of many different neurotransmitters, including glutamate (Van Harreveld and Fifkova, 1970; Fabricius et al., 1993; Molchanova et al., 2004; Enger et al., 2015). Indeed, spreading depolarization is the only process in the brain that causes a considerable increase in glutamate in the extracellular space. In non-ischemic tissue, glutamate increased up to 100 μM during spreading depolarization (Zhou et al., 2013). Accordingly, in ischemia *in vivo*, no glutamate increase is directly measurable before the onset of spreading depolarization, but the directly measurable glutamate increase follows the ischemia-induced spreading depolarization (Hinzman et al., 2015). Although the glutamate increase due to spreading depolarization does not occur in isolation but is accompanied by massive concentration changes of innumerable other factors, a pure glutamate application of 100 μM to primary cell culture is often presented as a proxy for ischemia (Randall and Thayer, 1992; Keelan et al., 1999; Vergun et al., 1999; Rueda et al., 2016). A blind spot in all these models is not only that the glutamate increase is studied in isolation from the many other changes, but also that molecular oxygen, which is, after all, absent during ischemia, is required not only for ATP production but also for numerous other cellular processes. To give but two examples: (1) Molecular oxygen is required for NO synthesis (Jiang et al., 2001; Uetsuka et al., 2002). So, how will an increase in extracellular glutamate cause increased NO production during ischemia (Keelan et al., 1999) when the normal NO synthesis is blocked by lack of molecular oxygen? How is it then that numerous review articles and neuroscience textbooks convey the message that ischemic cell injury is a consequence of excitotoxic increase in NO levels as a result of glutamate release (Belov Kirdajova et al., 2020)? Some NO production via endothelial NOS by reduction of nitrite may occur under anoxia (Gautier et al., 2006). However, this pathway is temporally limited because it depends on nitrite reserves, and it is questionable whether it is sufficient to maintain even the basal NO level. If anything, administration of an NO donor to the patient in acute ischemic stroke appears to have a beneficial effect if treatment is started within 6 h of the onset of ischemia (Woodhouse et al., 2015). (2) Another example is that the production of reactive oxygen species (ROS) also requires molecular oxygen (Dirnagl et al., 1995; Dreier et al., 1998; Peters et al., 1998). Thus, glutamate is a potent stimulus of ROS production in cell culture (Kubota et al., 2010), but the spreading depolarization-induced glutamate increase under ischemic conditions *in vivo* cannot cause an increase in ROS production because ROS production is blocked by lack of molecular oxygen (Dirnagl et al., 1995; Dreier et al., 1998; Peters et al., 1998). In fact, an increase in ROS production only occurs in the context of ischemia *in vivo*, if the tissue is fortunate enough to be reperfused and glutamate is taken up again into the cells from the extracellular space. That is, ROS production in response to an ischemic event *in vivo* increases to the pathological range only when (i) tissue is reperfused, (ii) neurons recover from the ischemia-induced spreading depolarization and (iii) extracellular glutamate concentration decreases again, which is the opposite of what is observed in cell culture upon glutamate application (Dirnagl et al., 1995; Dreier et al., 1998; Peters et al., 1998). Numerous studies on thrombolysis and mechanical recanalization of ischemic stroke patients have now shown that reperfusion does not lead to additional damage, but is currently the only chance for ischemic brain tissue to survive (Prabhakaran et al., 2015). Cell culture models are often touted as an effective substitute for animal experiments in order to reduce the number of animal experiments, and considerable political, economic and social pressure is built up, e.g., through poster campaigns in public transport, prizes and other initiatives, to achieve this goal. However, the above examples show how easily artifacts from cell culture experiments can corrupt scientific concepts, which is then reflected in countless review articles and textbook chapters, although the lack of validity of the concepts is easily revealed once they are tested *in vivo* (Dreier et al., 1998; Peters et al., 1998; Uetsuka et al., 2002), where, unlike in cell cultures, intracellular space is large and extracellular space is small, the full context of cell–cell, cell–circulation, and organ–organ interactions is preserved, spreading depolarizations occur, and neurons die from severe oxygen-glucose deprivation within minutes rather than within hours (Wappler et al., 2013). Cell culture experiments are of high scientific value for the study of intracellular signaling cascades only when used as a complement to, rather than as a replacement of, *in vivo* experiments, because *in vivo* experiments are indispensable for providing guidance and testing hypotheses derived from cell culture experiments, which are inevitably subject to myriad biases due to the artifact-laden circumstances.

## Conclusion

Spreading depolarization is a metastable universal reference state for acute CNS pathology. All relevant mechanisms in the context of acute ischemic or traumatic CNS injury, whether parenchymal, vascular, or immunologic, should be studied in relation to this reference state in order to correctly place them in the overall context. The fact that spreading depolarization can be harmless on the one hand and lead to neuronal death and even brain death on the other, that it is a huge electrochemical wave that is nevertheless difficult to detect in scalp EEG, that it can be triggered experimentally so easily and in a nicely stereotyped manner and yet is one of the most complex phenomena of the CNS with a highly complex pharmacology, explains at least in part why neuroscientists and neurologists have not struggled as hard with any other phenomenon for decades as they have with spreading depolarization.

## Author Contributions

CL and JD contributed substantially to the conception and design of the work and drafting and revising the manuscript for important intellectual content. JL, VH, CR, SM, NH, and JW drafted parts of the manuscript. All authors approved the final version to be published and agreed to be accountable for all aspects of the work.

## Conflict of Interest

The authors declare that the research was conducted in the absence of any commercial or financial relationships that could be construed as a potential conflict of interest. The reviewer OH declared a past co-authorship with the authors JD, CR to the handling editor.

## Publisher’s Note

All claims expressed in this article are solely those of the authors and do not necessarily represent those of their affiliated organizations, or those of the publisher, the editors and the reviewers. Any product that may be evaluated in this article, or claim that may be made by its manufacturer, is not guaranteed or endorsed by the publisher.

## Footnotes

1
www.cosbid.org

2
https://chem.libretexts.org/@go/page/21655

## Abbreviations

AC: alternating current
aSAH: aneurysmal subarachnoid hemorrhage
ACSF: artificial cerebrospinal fluid
[K^+^]_ACSF_: artificial cerebrospinal fluid with an elevated K^+^ concentration
BOLD: blood oxygen level-dependent
CNS: central nervous system
COSBID: Co-Operative Studies on Brain Injury Depolarizations
DCI: delayed cerebral ischemia
DW: diffusion-weighted
DC: direct current
ECoG: electrocorticography
EEG: electroencephalography
[Ca^2+^]_o_: extracellular Ca^2+^ concentration
[K^+^]_o_: extracellular K^+^ concentration
[Na^+^]_o_: extracellular Na^+^ concentration
FHM: familial hemiplegic migraine
IEE: ictal epileptic events
[K^+^]_i_: intracellular K^+^ concentration
[Ca^2+^]_i_: intraneuronal Ca^2+^ concentration
[Na^+^]_i_: intraneuronal Na^+^ concentration
IOS: intrinsic optical signal
MRI: magnetic resonance imaging
MCAO: middle cerebral artery occlusion
NUP: negative ultraslow potential
L-NNA: N^G^-nitro-L-arginine
NO: nitric oxide
NOS: nitric oxide synthase
ROS: reactive oxygen species
rCBF: regional cerebral blood flow
SPECT: single-photon emission computed tomography
SDEA: spreading depolarization with epileptiform activity
p_ti_O_2_: tissue partial pressure of oxygen
TIA: transient ischemic attack
TBI: traumatic brain injury.
