# Erratum: Migraine Aura, Transient Ischemic Attacks, Stroke, and Dying of the Brain Share the Same Key Pathophysiological Process in Neurons Driven by Gibbs–Donnan Forces, Namely Spreading Depolarization

**DOI:** 10.3389/fncel.2022.917669

**Published:** 2022-04-26

**Authors:** Frontiers Production Office

**Affiliations:** Frontiers Media SA, Lausanne, Switzerland

**Keywords:** migraine aura, traumatic brain injury, circulatory arrest, subarachnoid hemorrhage, spreading depolarization, spreading depression, brain death, brain ischemia

Due to a publisher error, the below statement was not included:

The reviewer OH declared a past co-authorship with the authors JD, CR to the handling editor.

The publisher apologizes for this mistake. The original version of this article has been updated.

